# Mitochondrial fission and bioenergetics mediate human lung fibroblast durotaxis

**DOI:** 10.1172/jci.insight.157348

**Published:** 2023-01-10

**Authors:** Ting Guo, Chun-sun Jiang, Shan-Zhong Yang, Yi Zhu, Chao He, A. Brent Carter, Veena B. Antony, Hong Peng, Yong Zhou

**Affiliations:** 1 Division of Pulmonary, Allergy and Critical Care Medicine, Department of Medicine, University of Alabama at Birmingham, Birmingham, Alabama, USA.; 2 Department of Respiratory Medicine, the Second Xiangya Hospital, Central-South University, Changsha, China.; 3 Birmingham Veterans Administration Medical Center, Birmingham, Alabama, USA.

**Keywords:** Cell Biology, Pulmonology, Cell migration/adhesion, Fibrosis, Mitochondria

## Abstract

Pulmonary fibrosis is characterized by stiffening of the extracellular matrix. Fibroblasts migrate in the direction of greater stiffness, a phenomenon termed durotaxis. The mechanically guided fibroblast migration could be a crucial step in the progression of lung fibrosis. In this study, we found primary human lung fibroblasts sense increasing matrix stiffness with a change of mitochondrial dynamics in favor of mitochondrial fission and increased production of ATP. Mitochondria polarize in the direction of a physiologically relevant stiffness gradient, with conspicuous localization to the leading edge, primarily lamellipodia and filopodia, of migrating lung fibroblasts. Matrix stiffness–regulated mitochondrial fission and durotactic lung fibroblast migration are mediated by a dynamin-related protein 1/mitochondrial fission factor–dependent (DRP1/MFF-dependent) pathway. Importantly, we found that the DRP1/MFF pathway is activated in fibrotic lung myofibroblasts in both human IPF and bleomycin-induced mouse lung fibrosis. These findings suggest that energy-producing mitochondria need to be sectioned via fission and repositioned in durotactic lung fibroblasts to meet the higher energy demand. This represents a potentially new mechanism through which mitochondria may contribute to the progression of fibrotic lung diseases. Inhibition of durotactic migration of lung fibroblasts may play an important role in preventing the progression of human idiopathic pulmonary fibrosis.

## Introduction

Fibroblasts are activated in response to injury and migrate to the wound site for tissue repair. Dysregulated fibroblast migration is associated with the development of pulmonary fibrosis (1). Understanding how lung fibroblasts migrate on the extracellular matrix (ECM) in their local tissue environment is a major question relevant to understanding the pathogenesis of pulmonary fibrosis. Lung fibrosis is characterized by stiffening of the ECM (2–5). Increasing matrix stiffness is not merely an epiphenomenon of fibrotic lung disease but is causally related to fibrosis progression (3, 5–12). Fibroblast migration can be directed by the rigidity of the microenvironment, i.e., from regions of lower to higher stiffness, a process known as durotaxis (13). Durotactic fibroblast migration contributes to both physiological and pathological processes ranging from development to cancer progression (14, 15). Durotaxis can lead to recruitment of pathological fibroblasts to the area of established fibrosis, thereby contributing to the progression of lung fibrosis. Previous studies have revealed the distinctive roles of focal adhesion and cytoskeletal dynamics in fibroblast durotaxis (16). Although these represent crucial aspects of the mechanisms underlying durotaxis, we remain far from having a comprehensive understanding of the mechanically guided cell migration.

Mitochondria are highly dynamic organelles that involve a variety of central metabolic processes. Continuous fission and fusion play a critical role in mitochondrial network organization, linking mitochondrial structure to function (17). The highly dynamic and functionally versatile mitochondria make them important stress sensors, which allow for cellular adaptation to the environment (18). Emerging evidence suggests that mitochondria are central mediators of cellular responses to extracellular mechanical signals. A recent study found that ECM stiffness influences baseline metabolism in cardiac myocytes, suggesting that mitochondria are an integral part of the cell’s mechanosensing apparatus (19). Currently, it is not known whether ECM stiffness regulates mitochondrial structure and function, particularly in the context of durotactic fibroblast migration and pulmonary fibrosis.

To gain further insight into the mechanisms underlying fibroblast durotaxis, we investigate the potential effects of matrix stiffness on mitochondrial dynamics, ATP production, and cell migration in primary human lung fibroblasts. We generate polyacrylamide (PA) hydrogels with a stiffness gradient simulating the mechanical environment of the physiological lung. Utilizing the stiffness gradient PA gels, we observe mitochondrial distribution in migrating lung fibroblasts and identify key molecules and signaling pathways involved in regulation of mitochondrial function during lung fibroblast durotaxis. To evaluate the relevance of our in vitro findings to lung fibrosis, we analyze expression of the identified key regulators of mitochondrial function and durotaxis in (myo)fibroblasts in both human idiopathic pulmonary fibrosis (IPF) and fibrotic mouse lungs as well as their normal control lungs.

## Results

### Stiff matrix promotes mitochondrial fission in primary human lung fibroblasts.

**Figure 1:**
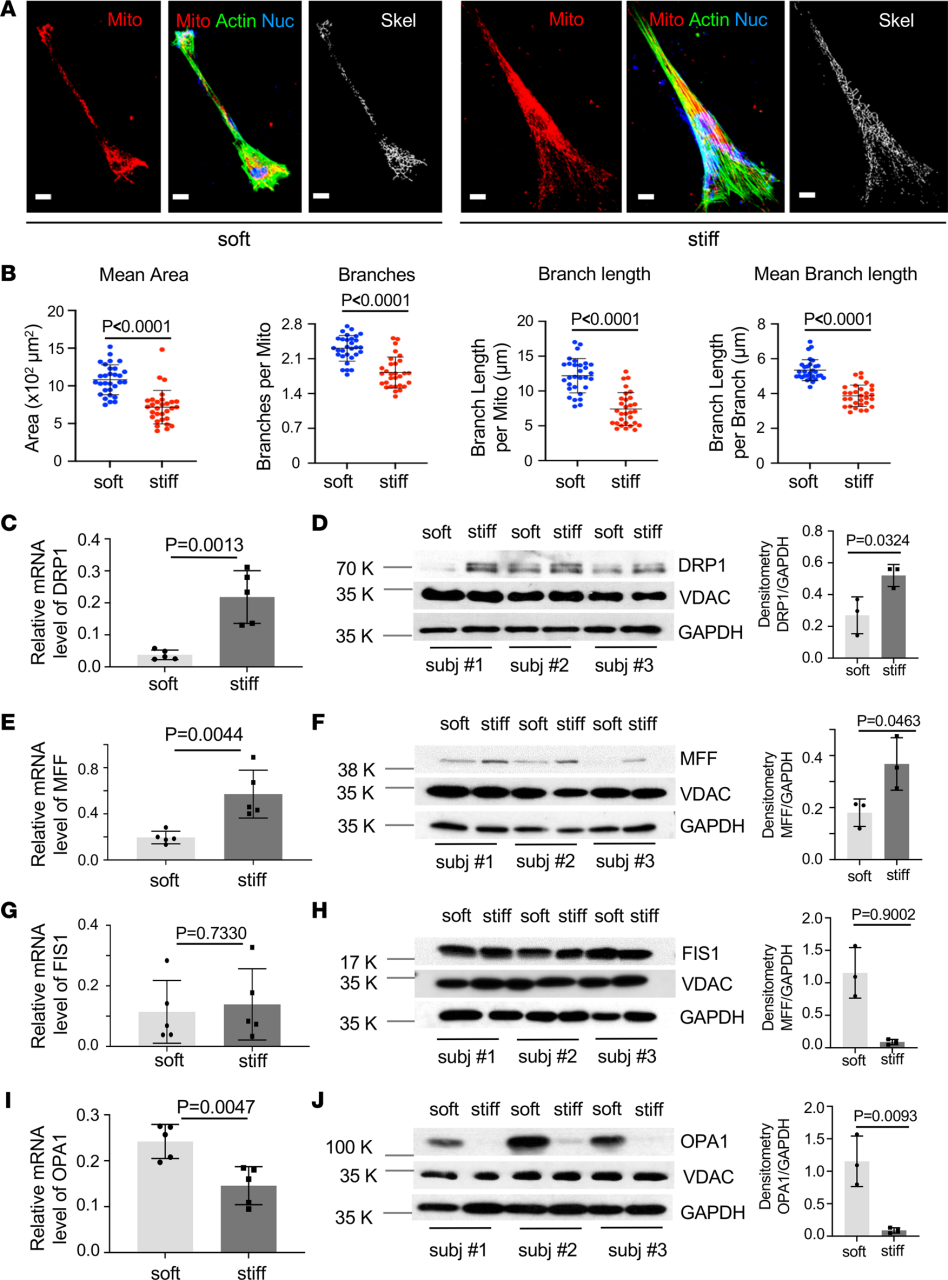
Stiff matrix promotes mitochondrial fission in primary human lung fibroblasts. Primary human lung fibroblasts were cultured on soft and stiff PA gels for 48 hours. (**A**) Mitochondria and actin filaments were stained by MitoTracker (red) and phalloidin (green). The binarized mitochondria were converted into topological skeletons. Scale bar = 20 μm. (**B**) The mean mitochondrial area, number of branches, length of branches, and mean branch length in the skeletonized mitochondrial network were measured by Mitochondria Analyzer plugin in ImageJ (NIH). Bar graphs represent mean ± SD per cell from 30 cells derived from 3 human participants (*n* = 10 cells per participant) under each condition. (**C**–**J**) Relative mRNA levels of DRP1 (**C** and **D**), MFF (**E** and **F**), FIS1 (**G** and **H**), and OPA1 (**I** and **J**) were determined by quantitative reverse transcription PCR. GAPDH was used as an internal reference control. Protein levels of DRP1, MFF, FIS1, and OPA1 were determined by immunoblot. GAPDH and VDAC were used as loading controls. Densitometry was performed using ImageJ. Bar graphs represent mean ± SD of experiments from 3–5 independent human participants, each performed in triplicates. A 2-tailed Student’s *t* test was used for comparison between groups.

To determine effects of matrix stiffness on mitochondrial dynamics, primary human lung fibroblasts isolated from donors were cultured on soft (1 kPa) and stiff (20 kPa) PA gels that mimic the stiffness grades of normal and fibrotic lungs (2–5). We observed that lung fibroblasts cultured on stiff matrix substrates had a significant decrease in the mean mitochondrial area, a measure of mitochondrial network integrity (20), compared with cells cultured on soft matrix substrates (Figure 1, A and B), suggesting that stiff matrix reduces the integrity of mitochondria in human lung fibroblasts. Evaluation of the connectivity of the mitochondrial network by skeletonization analysis revealed that stiff matrix decreased the number of mitochondrial branches, the length of branches per mitochondrion, and the mean branch length compared with soft matrix (Figure 1, A and B). Furthermore, human lung fibroblasts cultured on stiff matrix displayed stronger mitochondrial signals, evaluated by staining of mitochondria followed by flow cytometric analyses, than cells cultured on soft matrix (Supplemental Figure 1; supplemental material available online with this article; https://doi.org/10.1172/jci.insight.157348DS1). Together, these data indicate that stiff matrix conditions favor fragmentation of the mitochondrial network.

Dynamin-related protein 1 (DRP1), a member of the dynamin family of GTPases, is the main player in mitochondrial fission (21). Additionally, mitochondrial fission is regulated by integral outer membrane proteins, such as fission 1 (FIS1) and mitochondrial fission factor (MFF), that are required for activation of cytoplasmic DRP1 and its recruitment to the mitochondrial fission site (22, 23). In contrast, mitochondrial fusion is orchestrated by factors such as optic atrophy 1 (OPA1), which helps fuse the mitochondrial membranes and facilitate the formation of contiguous mitochondrial chains (24). We found that human lung fibroblasts cultured on stiff matrix expressed significantly higher levels of DRP1 and MFF at the mRNA and protein levels than cells cultured on soft matrix (Figure 1, C–F). In contrast, FIS1 expression was equivalent between 2 different stiffness conditions (Figure 1, G and H). Meanwhile, stiff matrix significantly decreased the mRNA and protein expression of OPA1 compared with soft matrix (Figure 1, I and J). Taken together, these data suggest that stiff matrix alters mitochondrial dynamics in favor of mitochondrial fission by a DRP1/MFF-dependent pathway in primary human lung fibroblasts.

### Mitochondrial fission is associated with matrix stiffness–regulated human lung fibroblast migration.

**Figure 2:**
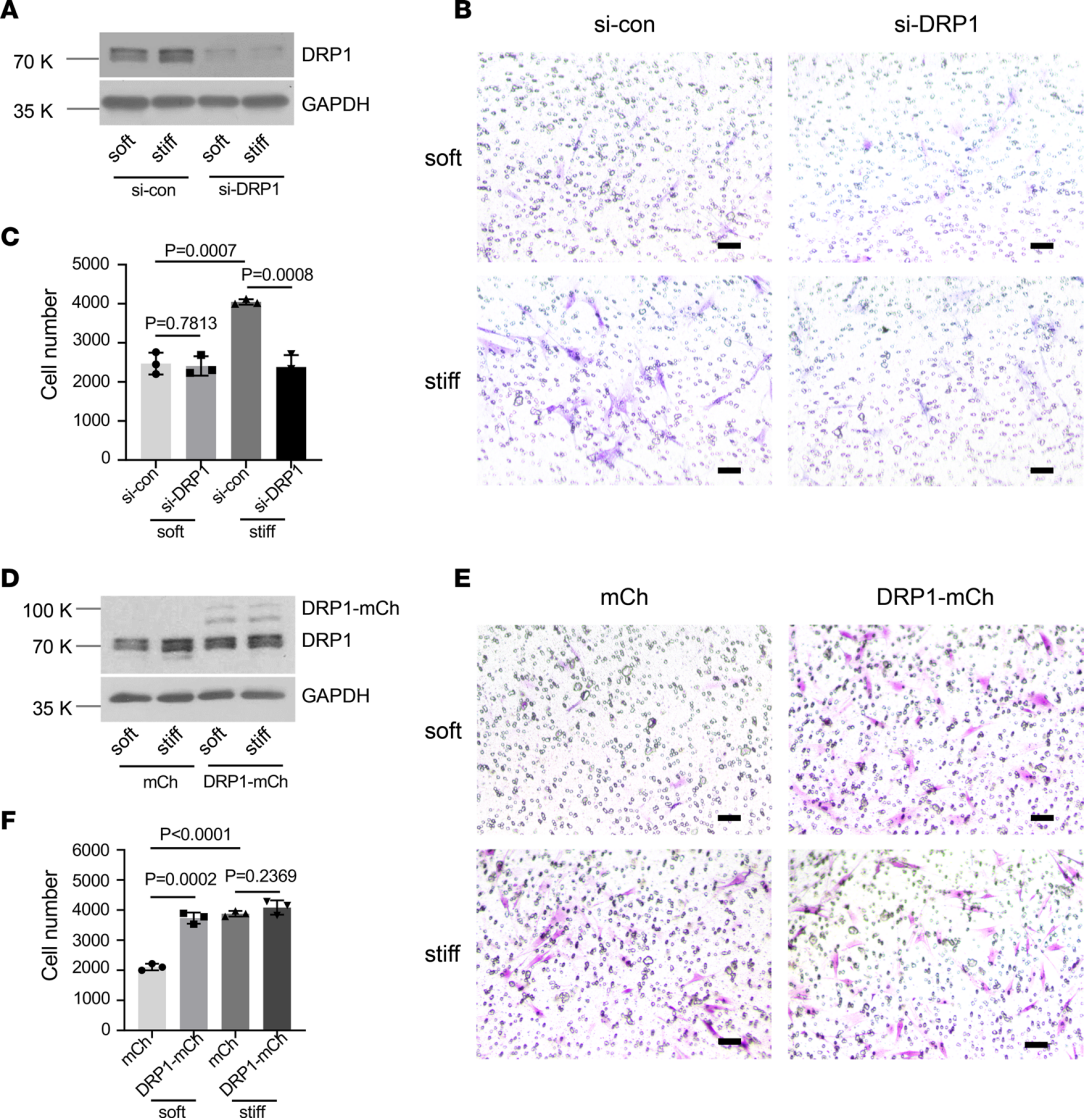
Mitochondrial fission regulates matrix stiffness–dependent migration of human lung fibroblasts. (**A**) DRP1-specific siRNA (si-DRP1) and the control siRNA (si-con) were transfected into human lung fibroblasts. Knockdown of DRP1 expression was determined by immunoblot. GAPDH was used as loading control. (**B** and **C**) Lung fibroblasts transfected with si-DRP1 and the control siRNA (si-con) were cultured on soft and stiff hydrogels for 48 hours. Cells were removed from the gels, and an equal number of living cells were plated in Transwell inserts. After 7 hours, migrating cells were stained with crystal violet and were quantified as described in Supplemental Methods. (**D**) Plasmids expressing mCherry-tagged DRP1 (DRP1-mCh) and mCherry alone (mCh) were transfected into human lung fibroblasts. Overexpression of DRP1 was determined by immunoblot with anti-DRP1 antibody. GAPDH was used as loading control. (**E** and **F**) Lung fibroblasts transfected with DRP1-mCh and mCh were cultured on soft and stiff hydrogels for 48 hours. Cells were detached and transferred to Transwells. Cell migration assay was determined as described above. Bar graphs represent mean ± SD of 3 independent experiments. Similar results were obtained with repeated experiments on 3 lung fibroblast isolates. A 2-tailed Student’s *t* test was used for comparison between groups. Scale bar = 50 μm.

To investigate the potential role of mitochondrial fission in matrix stiffness–regulated human lung fibroblast migration, we performed siRNA-mediated DRP1 knockdown in primary human lung fibroblasts (Figure 2A). Knockdown of DRP1 expression significantly increased mitochondrial connectivity on stiff matrix (Supplemental Figure 2), consistent with reduced mitochondrial fission. Boyden chamber migration assays showed that knockdown of DRP1 blocked stiff matrix–induced human lung fibroblast migration (Figure 2, B and C). Knockdown of DRP1 expression also increased mitochondrial connectivity on soft matrix. However, this change was not statistically significant (Supplemental Figure 2). Knockdown of DRP1 did not alter lung fibroblast migration under soft matrix conditions (Figure 2, B and C). Conversely, overexpression of DRP1 significantly reduced mitochondrial connectivity on soft matrix (Supplemental Figure 3) and significantly increased lung fibroblast migration under soft matrix conditions (Figure 2, D–F). Overexpression of DRP1 had no significant effects on mitochondrial connectivity and cell migration under stiff matrix conditions, presumably due to a low efficiency of exogenous DRP1 expression under stiff matrix conditions where endogenous DRP1 expression was elevated. Additionally, human lung fibroblasts preconditioned on soft versus stiff matrix did not differ in cell attachment and viability when reseeded into Transwells (Supplemental Figure 4). Together, these data suggest that DRP1-mediated mitochondrial fission is associated with matrix stiffness–regulated human lung fibroblast migration.

### Fabrication and characterization of stiffness gradient PA hydrogels simulating the rigidity of physiological lungs.

Fibrotic lung tissues are characterized by stiffening of lung parenchyma, resulting in spatial gradients between focal “peaks” and “valleys” of matrix stiffness (25). By modifying a previously published, straightforward approach (26), we generated PA gels with a stiffness gradient simulating the fibrotic lungs (Supplemental Figure 5A). In this method, a first PA gel of varying thickness was prepolymerized. Then a second PA gel was polymerized on top. The differential diffusion of acrylamide monomers and crosslinkers across the varying thickness of the prepolymerized gel created a stiffness gradient on the second gel. To characterize the stiffness gradient PA gels, we measured Young’s moduli of the compound gels parallel to the ramp axis by atomic force microscopy (AFM). Data showed that the average stiffness values in the direction of predicted stiffness gradient were 0.15 ± 0.08 kPa (median ± SD) at 1 mm region, 0.70 ± 0.24 kPa at 5 mm region, 1.99 ± 0.52 kPa at 10 mm region, 7.20 ± 1.31 kPa at 15 mm region, and 16.07 ± 2.17 kPa at 20 mm region (Supplemental Figure 5, B and C). The average gradient strength (i.e., the degree of stiffness change per length) was 0.83 kPa/μm (Supplemental Figure 5D). The characteristics of stiffness gradient PA gels are similar to the mechanical properties of fibrotic lungs previously measured by others and us (2–5).

### Polarized mitochondrial distribution is associated with human lung fibroblast durotaxis.

**Figure 3:**
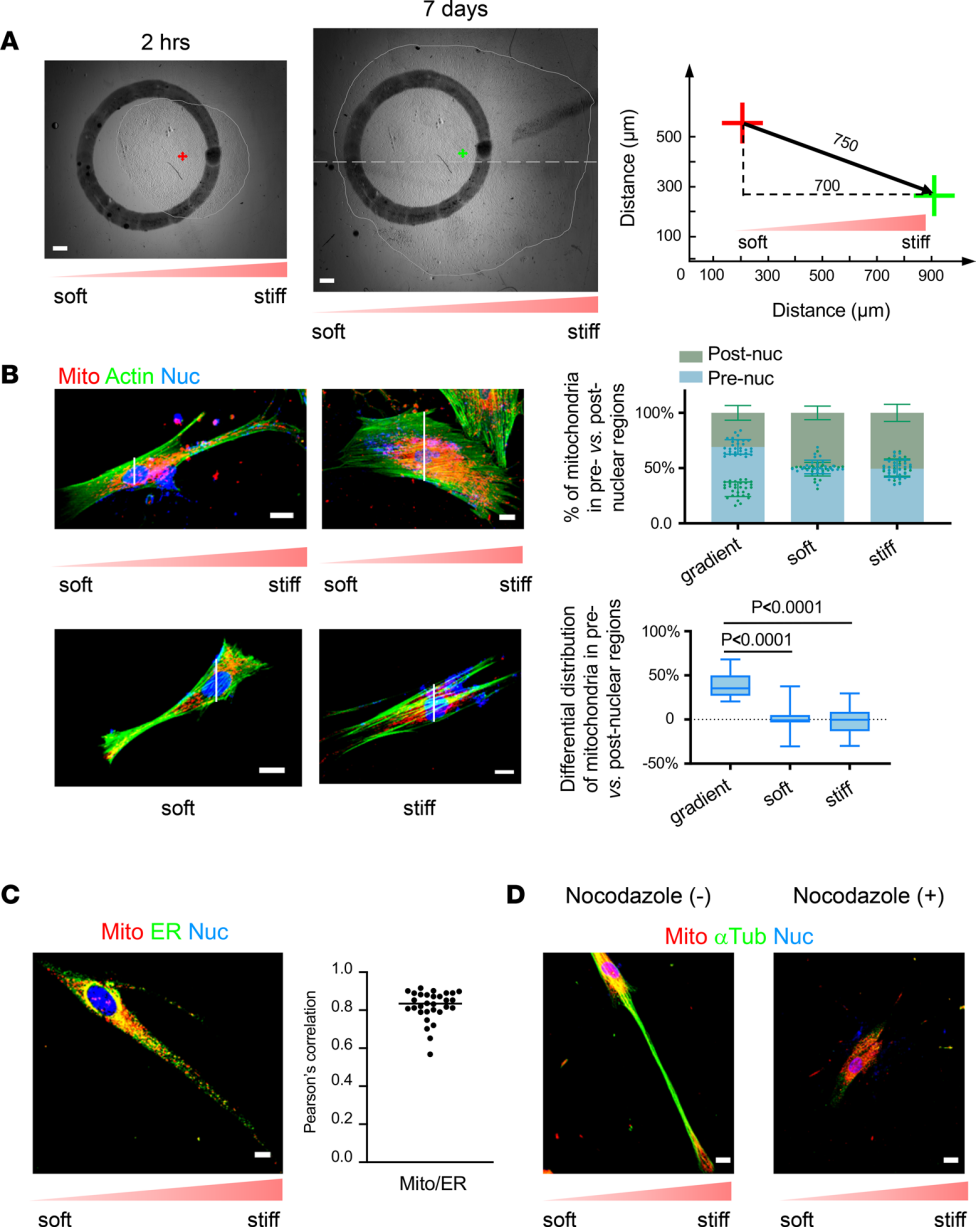
Redistribution of mitochondria in lamellipodia/filopodia is associated with human lung fibroblast durotaxis. (**A**) Human lung fibroblasts were initially seeded at ~2 kPa region (circle) on stiffness gradient gels. Cells completely attached to the gels after 2 hours. Cells were allowed to migrate on stiffness gradient gels for 7 days. The overall distance of cell migration (750 μm) was the distance between the centroids of the cell population at 2 hours (red cross) and 7 days (green cross). The distance of durotactic migration (700 μm) was defined as the distance of migration in the direction of the stiffness gradient. Scale bar = 500 μm. (**B**) Human lung fibroblasts cultured on stiffness gradient gels or gels with uniform stiffness (soft and stiff) were stained with MitoTracker (red) and phalloidin (green). Nuclei were stained with DAPI (blue). Cell body was divided into pre- (right) and postnuclear (left) regions. Mitochondrial distribution in pre- and postnuclear regions was quantified as the percentage of mitochondria in each region. Bar graphs represent mean ± SD per cell from 30 cells derived from 3 human participants (*n* = 10 cells per participant) under each condition. Box plots show the interquartile range (box), median (line), and minimum and maximum (whiskers). Mitochondrial polarization was evaluated by differential distribution of mitochondria in pre- versus postnuclear regions. Statistical analysis was performed by 1-way ANOVA. Scale bar = 20 μm. (**C**) Human lung fibroblasts migrating on stiffness gradient gels were stained with MitoTracker (red) and ER Tracker (green). Nuclei were stained with DAPI (blue). Colocalization of mitochondria and ER from 30 individual cells was quantified by Pearson’s correlation coefficient analysis. Scale bar = 20 μm. (**D**) Human lung fibroblasts were cultured on stiffness gradient gels in the presence or absence of nocodazole. Cells were stained with MitoTracker (red) and anti–α-tubulin antibody (green). Nuclei were stained with DAPI (blue). Scale bar = 20 μm.

Previous studies have shown that human fibroblasts display the strongest durotactic response when migrating on the stiffness gradient in a 2–7 kPa range (15). We seeded primary human lung fibroblasts at the 10 mm region (1.99 ± 0.52 kPa) in the stiffness gradient PA gels and observed cell migration for 7 days. The fibroblast population moved an overall distance of 750 μm toward the stiffer region, in which the distance in the direction of increasing stiffness (durotactic migration) was 700 μm (Figure 3A). To rule out the potential effect of stiff matrix–induced lung fibroblast proliferation on cell population–based durotactic assay, we performed time-lapse microscopy to track single–lung fibroblast movement on stiffness gradient hydrogels for 24 hours (Supplemental Figure 6A and Supplemental Video 1). We observed that the distance of single–lung fibroblast durotactic migration was 231 μm (Supplemental Figure 6, B and C). Together, these results demonstrated the durotactic migration of human lung fibroblasts on a physiologically relevant stiffness gradient.

Fibroblast migration requires globular actin incorporation into the barbed end of polymerized actin filaments and formation of cellular protrusions (27). We observed that durotactic lung fibroblasts formed filopodia (thin and long cytoplasmic protrusions) and lamellipodia (flat and large projections of cell membrane) at the leading edge of the cell body on the stiffer side of stiffness gradient PA gels (Figure 3B). Remarkably, mitochondria were found to be more frequently located at the leading edge of durotactic lung fibroblasts, primarily in the filopodia and lamellipodia. Furthermore, mitochondria were associated with actomyosin where the ATP-consuming cell motor localizes (Figure 3B). The endoplasmic reticulum (ER) is involved in constriction of mitochondria during fission (28). We found that the polarized mitochondria were closely associated with the ER in durotactic lung fibroblasts (Figure 3C), consistent with the previous findings that matrix stiffness regulates mitochondrial fission (Figures 1 and 2). The microtubule cytoskeleton plays an important role in the long-distance transport and positioning of mitochondria (29). We observed that the majority of mitochondria in durotactic lung fibroblasts aligned along the microtubule cytoskeleton. Disturbance in microtubule integrity by nocodazole, a microtubule-depolymerizing drug, triggered disarray of polarized mitochondria on stiffness gradient substrates (Figure 3D). These results demonstrated that the polarized mitochondrial distribution in durotactic lung fibroblasts is dependent upon intact microtubules.

### DRP1/MFF-dependent mitochondrial fission and bioenergetics mediate human lung fibroblast durotaxis.

**Figure 4:**
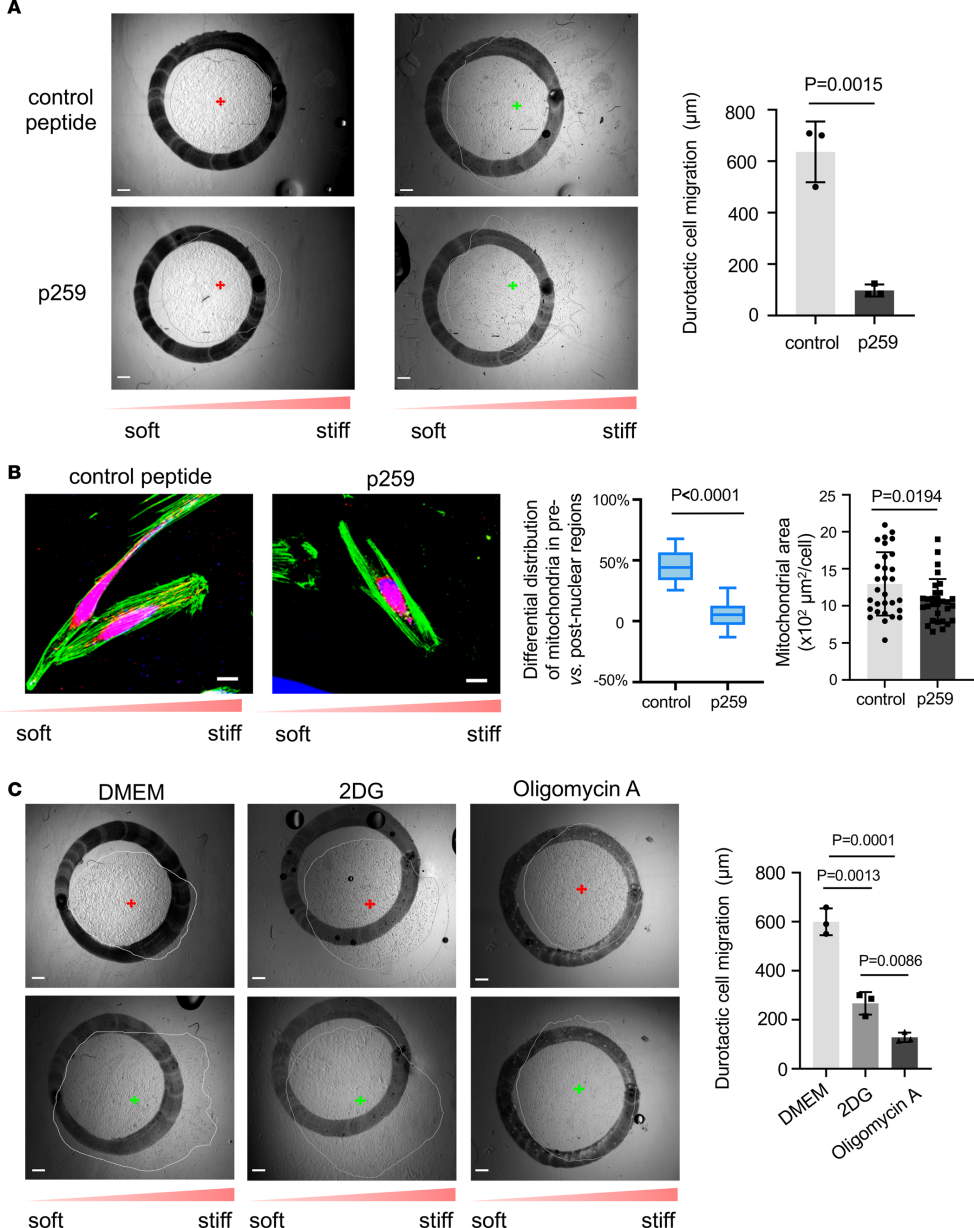
Mitochondrial fission and bioenergetics mediate human lung fibroblast durotaxis. (**A**) Human lung fibroblasts were cultured on stiffness gradient gels in the presence of P259 or TAT carrier control peptides. Durotactic cell migration was determined as described in Figure 3. Bar graphs represent mean ± SD of 3 separate experiments. Similar results were obtained from 3 lung fibroblast isolates. A 2-tailed Student’s *t* test was used for comparison between groups. Scale bar = 500 μm. (**B**) Mitochondria and actin filaments were stained by MitoTracker (red) and phalloidin (green). Mitochondrial distribution and area were determined as described previously. Results are the means ± SD from 30 individual cells derived from 3 human participants (*n* = 10 cells per participant) under each condition. Box plots show the interquartile range (box), median (line), and minimum and maximum (whiskers). Statistical analysis was performed by 1-way ANOVA. Scale bar = 20 μm. (**C**) Human lung fibroblasts were cultured on stiffness gradient gels in the presence or absence of 2-DG or oligomycin A. Durotactic cell migration was determined as described previously. Bar graphs represent mean ± SD of 3 separate experiments. Similar results were obtained from 3 lung fibroblast isolates. Statistical analysis was performed by 1-way ANOVA. Scale bar = 500 μm.

Since our data suggest that DRP1 and probably MFF may mediate matrix stiffness–dependent mitochondrial fission and human lung fibroblast migration (Figures 1 and 2), we next determined the role of the DRP1/MFF pathway in durotactic lung fibroblast migration. P259 is an antagonist peptide that functions to inhibit mitochondrial fission by specifically blocking the interaction of DRP1 and MFF (30). The conjugation of a cell-permeating TAT carrier peptide enables effective delivery of P259 through the plasma membrane (30, 31). We found that P259-treated human lung fibroblasts migrated an average distance of 98 ± 23 μm in the direction of increasing stiffness, whereas the TAT control peptide–treated cells migrated an average distance of 636 ± 118 μm (Figure 4A). Similar results were obtained by tracking single–lung fibroblast migration on stiffness gradient PA gels (Supplemental Figure 8A and Supplemental Videos 2 and 3). These data indicate that P259 treatment significantly inhibited mechanically guided lung fibroblast migration. Furthermore, P259-treated lung fibroblasts showed reduction in the amount of mitochondria, lesser mitochondrial polarization, and loss of typical structures of filopodia and lamellipodia (Figure 4B).

A major function of mitochondria is the conversion of nutrients into energy in a form of ATP for different cellular needs. Cell migration is known as an energy-intensive process (32). Consistent with this, we observed that stiff matrix promoted ATP production by human lung fibroblasts compared with soft matrix (Supplemental Figure 7). Given that filopodia and lamellipodia are the energy-consuming structures responsible for pulling the cell forward (33), polarized distribution of mitochondria and increased ATP production can meet the increasing energy demands for durotactic migration of human lung fibroblasts. P259 treatment did not change ATP production by human lung fibroblasts under either soft or stiff matrix conditions (Supplemental Figure 7), suggesting that stiff matrix–induced ATP production is independent of mitochondrial fission. To determine specific bioenergetic processes involved in human lung fibroblast durotaxis, we cultured cells on stiffness gradient PA gels in the presence or absence of 2-deoxyglucose (2-DG), a glycolysis inhibitor, or oligomycin A, a potent ATP synthase inhibitor that has been shown to suppress oxidative phosphorylation (OXPHOS). We found that both 2-DG and oligomycin A treatments significantly inhibited human lung fibroblast durotaxis (Figure 4C; Supplemental Figure 8, B and C; and Supplemental Videos 4–7). However, oligomycin A showed a greater inhibitory effect than 2-DG (Figure 4C). Together, these data suggest that DRP1/MFF-dependent mitochondrial fission and bioenergetics mediate durotactic lung fibroblast migration.

### Expression of DRP1 and MFF is elevated in lung myofibroblasts in both human IPF and bleomycin injury–induced mouse lung fibrosis.

**Figure 5:**
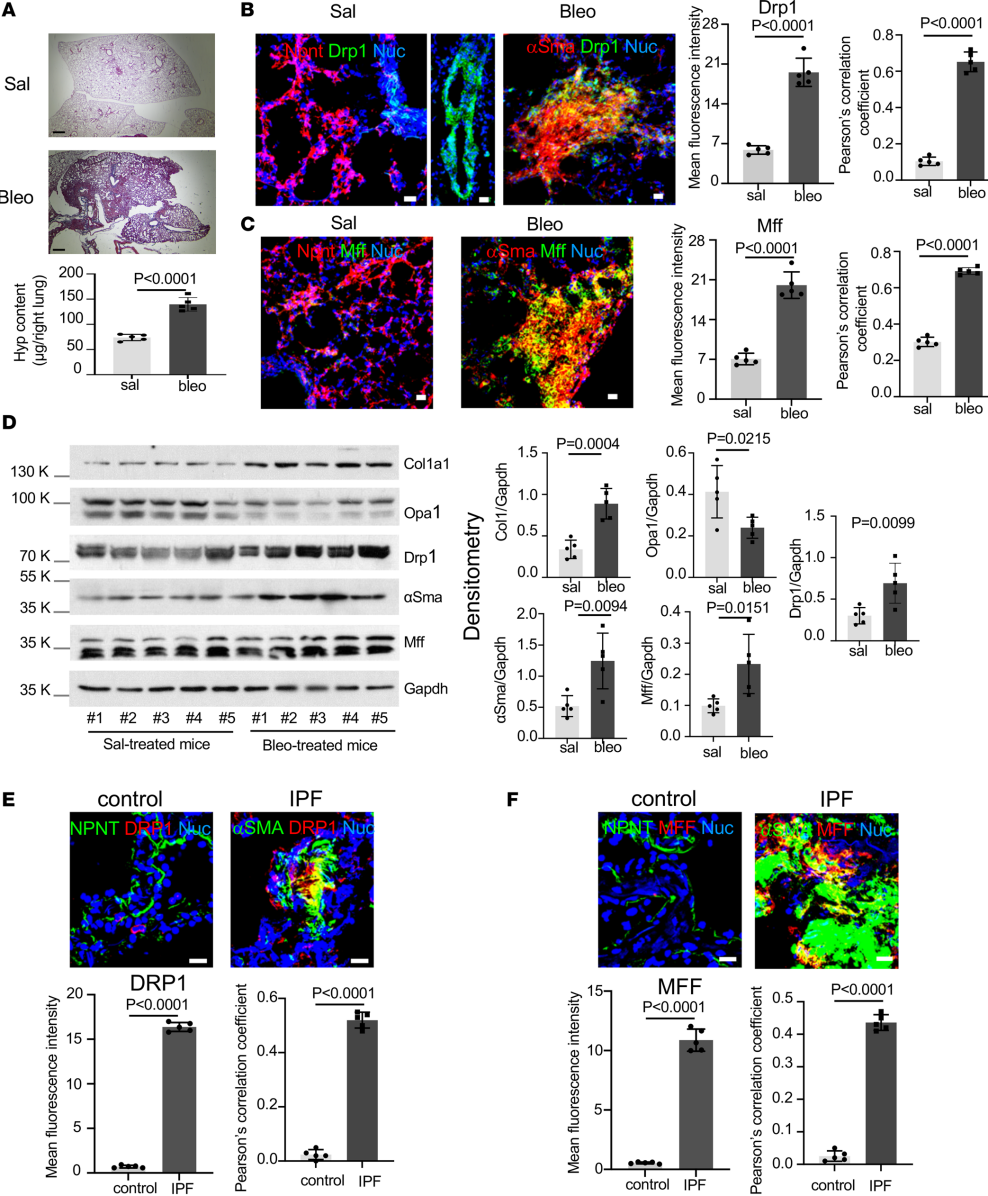
Expression of DRP1 and MFF is elevated in lung myofibroblasts in both human IPF and bleomycin injury–induced experimental lung fibrosis in mice. (**A**) C57BL/6 mice were administered 2 U/kg bleomycin (Bleo) or an equal volume of saline (Sal). Lung tissues were harvested at 21 days. Collagen deposition in mouse lungs was evaluated by Masson’s trichrome stain and hydroxyproline content assays. Scale bar = 500 μm. (**B** and **C**) Expression of Drp1 (**B**) and Mff (**C**) in normal fibroblasts (marked by Npnt expression) and fibroblasts (marked by α-Sma expression) in saline- and bleomycin-treated mouse lungs was evaluated by confocal immunofluorescence microscopy. Fluorescence intensities are mean ± SD of randomly selected areas from 5 mice under each condition. Colocalization of Drp1/Mff expression with (myo)fibroblasts was evaluated by Pearson’s correlation coefficient analyses. Scale bar = 20 μm. (**D**) Protein levels of Col1a1, α-Sma, Drp1, Mff, and Opa1 in saline-treated and bleomycin-treated mouse lungs were determined by immunoblot. GAPDH was used as loading control. Relative density was normalized to GAPDH. Bar graphs represent mean ± SD from 5 mice in each group. (**E** and **F**) Expression of DRP1 (**E**) and MFF (**F**) in normal fibroblasts and myofibroblasts in human IPF and normal control lungs was evaluated by confocal immunofluorescence microscopy. Nuclei were stained with DAPI (blue). Fluorescence intensities are mean ± SD of randomly selected areas from 5 mice under each condition. Colocalization of DRP1/MFF expression with (myo)fibroblasts was evaluated by Pearson’s correlation coefficient analyses. Scale bar = 20 μm; a 2-tailed Student’s *t* test was used for comparison between groups.

**Figure 6:**
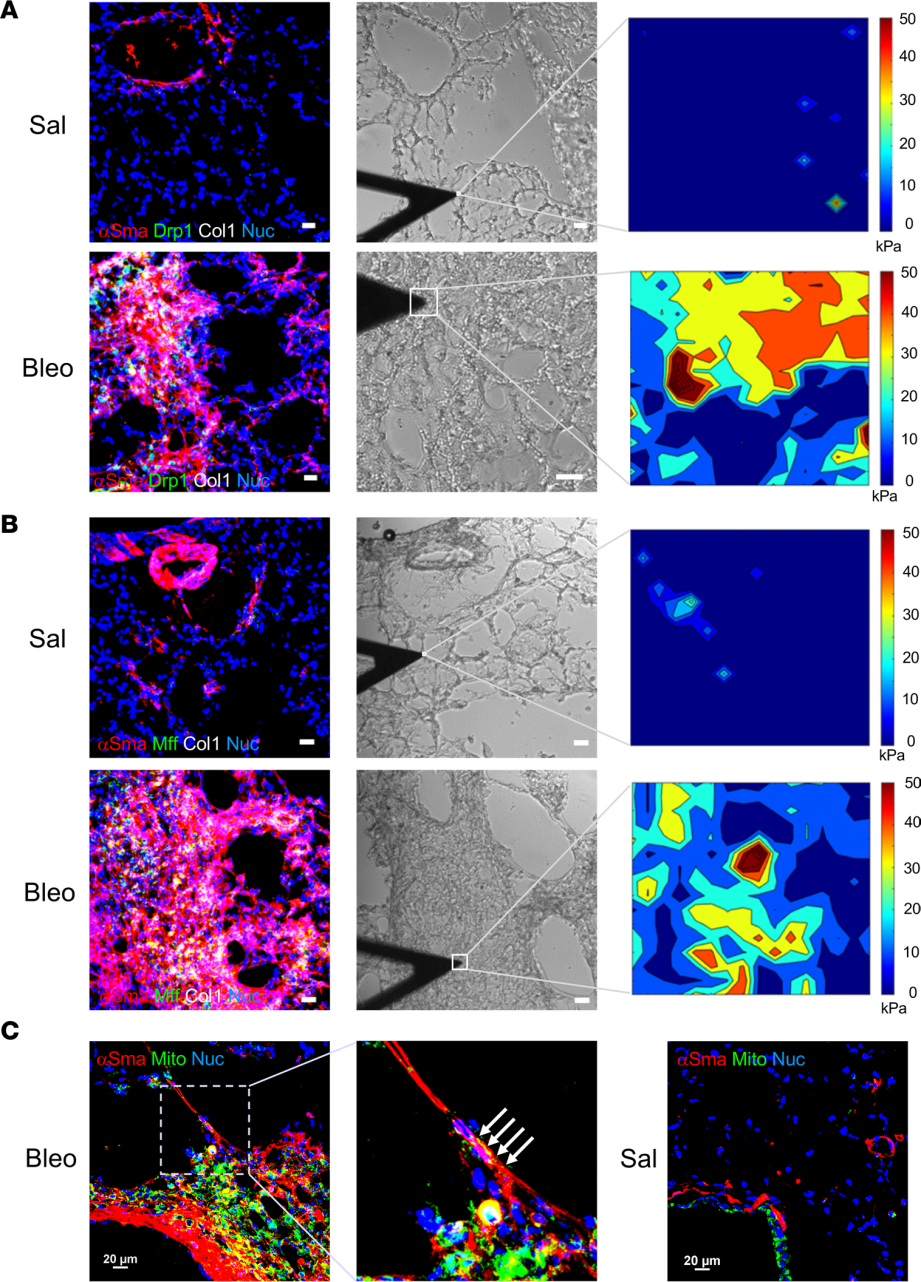
Matrix stiffness–regulated expression of Drp1 and Mff and polarization of mitochondria in lung myofibroblasts are evidenced in bleomycin-induced mouse lung fibrosis. (**A** and **B**) Frozen lung tissue sections from saline- or bleomycin-treated mice were costained by anti-Drp1 (**A**), anti-Mff (**B**) (green), anti–α-Sma (red), and anti–collagen I (white) antibodies. Nuclei were stained with DAPI (blue). The mechanical properties of the fibrotic lung areas (20 μm × 20 μm) and normal alveolar areas (2 μm × 2 μm) were determined by AFM microindentation on adjacent lung tissue sections (boxed regions in the middle panels). Values of lung tissue stiffness were shown by heatmaps. Scale bar = 20 μm. (**C**) Mouse lung tissue sections were stained by anti–α-Sma antibody (red) and MitoTracker (green). Nuclei were stained with DAPI (blue). Prenuclear localization of mitochondria (arrows) toward the stiffened fibrotic region was seen in a myofibroblast at the periphery of lung fibrosis. Scale bar = 20 μm.

To evaluate whether the DRP1/MFF-dependent fission pathway is activated in lung (myo)fibroblasts in experimental lung fibrosis, we treated wild-type C57BL/6 mice with bleomycin via oropharyngeal aspiration. Bleomycin treatment resulted in substantial deposition of collagens in mouse lungs (Figure 5A), indicating lung fibrosis. Confocal immunofluorescence microscopy showed that normal lung fibroblasts identified by expression of Npnt, a newly discovered marker of alveolar fibroblasts (34), in saline-treated mice expressed little Drp1 and Mff, whereas fibrotic (myo)fibroblasts identified by expression of α–smooth muscle actin (α-Sma) in bleomycin-treated mice expressed high levels of Drp1 and Mff (Figure 5, B and C, and Supplemental Figure 9, A–D). Drp1-positive signals were also present on the walls of blood vessels and large airways in mouse lungs. Consistent with these data, immunoblot analysis showed that the protein levels of Drp1 and Mff, along with type I procollagen and α-Sma, were significantly increased in bleomycin-treated mouse lungs as compared with saline-treated mouse lungs (Figure 5D). In contrast, the protein level of Opa1 was decreased in bleomycin- versus saline-treated mice. Confocal immunofluorescence microscopy demonstrated increased expression of DRP1 and MFF in α-SMA–positive myofibroblasts and fibroblasts within the fibroblastic foci in human IPF lungs, but not in normal alveolar fibroblasts in the control lungs (Figure 5, E and F, and Supplemental Figure 9, E–H). These data indicate that the DRP1/MFF-dependent mitochondrial fission pathway is activated in both human IPF and experimental pulmonary fibrosis in mice. Mechanical testing by AFM demonstrated that increased expression of Drp1 and Mff was associated with tissue stiffening in a bleomycin-induced mouse model of lung fibrosis (Figure 6, A and B). Furthermore, we captured an α-SMA–positive myofibroblast that appeared to distribute the fragmented mitochondria toward the stiffened fibrotic area while it migrated from the relatively normal region to the fibrotic region (Figure 6C). Additionally, lung myofibroblasts in the fibrotic region presented much stronger mitochondrion-positive signals compared with smooth muscle cells and Npnt-positive alveolar fibroblasts in the control lungs. These observations are consistent with the in vitro findings that mitochondrial fission mediates fibroblast durotaxis in lung fibrosis.

## Discussion

There is a strong correlation between stiffening of the lung microenvironment and activation of lung fibroblasts (3, 5–12), the main effector cells in pulmonary fibrosis. Emerging evidence suggests that mitochondrial dysfunction is a key player in the pathogenesis of human IPF (35). In this study, we identified mitochondria as a critical mechanotransducer at the intersection between extracellular mechanical signals and lung fibroblast durotaxis. We found that primary human lung fibroblasts sense mechanical signals from matrix substrates with changing mitochondrial dynamics in favor of mitochondrial fission and increasing ATP production. Lung fibroblasts reposition their mitochondrial distribution toward the stiffer region on stiffness gradient matrix substrates, mimicking the mechanical properties of fibrotic lungs. We propose that energy-producing mitochondria need to be sectioned via fission and repositioned in durotactic lung fibroblasts to meet the higher energy demand. We demonstrated that matrix stiffness–regulated mitochondrial fission and human lung fibroblast migration are mediated by a DRP1/MFF-dependent pathway. Importantly, we provided evidence that both DRP1 and MFF expression are elevated in association with lung tissue stiffening in human IPF and/or bleomycin-induced mouse lung fibrosis, suggesting mechanical activation of the DRP1/MFF pathway in pulmonary fibrosis in vivo. The current study reveals a potentially new role of mitochondria in modulating the migratory and invasive phenotype of lung fibroblasts and the progression of pulmonary fibrosis.

There have been limited studies on the role of mitochondrial dynamics in fibroblasts and pulmonary fibrosis. A previous study reported that astaxanthin, a carotenoid which is used as a dietary supplement, promotes DRP1-dependent mitochondrial fission in lung fibroblasts, whereas profibrotic cytokine TGF-β1 does not. Fibrotic fibroblasts display enhanced mitochondrial fission, leading to a pool of dysfunctional mitochondria (36, 37). In the current study, we found that mitochondrial network remodeling was important in durotactic lung fibroblast migration. DRP1-dependent mitochondrial fission mediated mechanically guided lung fibroblast migration. Mitochondrial fission is a highly regulated process that enables both biogenesis of new mitochondria and the clearance of dysfunctional mitochondria (37, 38). A recent study has discovered 2 functionally and mechanistically distinct types of mitochondrial fission, both mediated by DRP1 (39). One type involves DRP1/MFF-mediated mitochondrial division at the midzone that leads to the proliferation of mitochondria. The other is mediated by DRP1/FIS1 at the periphery that enables the damaged mitochondria destined for mitophagy. Our data show that stiff matrix upregulated expression of DRP1 and MFF, but not FIS1, in human lung fibroblasts. Stiff matrix–induced expression of DRP1 and MFF promoted mitochondrial fission in lung fibroblasts. Furthermore, blockade of the interaction between DRP1 and MFF inhibited human lung fibroblast durotaxis. These findings suggest that the DRP1/MFF pathway mediated stiff matrix–induced mitochondrial fission, which promoted durotactic lung fibroblast migration. Since levels of FIS1 expression are equivalent under soft and stiff matrix conditions, we do not expect that matrix stiffening would lead to expelling a large amount of mitochondria in migrating lung fibroblasts.

Cells can sense both biochemical and biophysical cues in the microenvironment, triggering changes in cytoskeleton organization and contractility for cell migration (40). In migrating cells, actin polymerization and actomyosin-based activity create mechanical forces and consume a significant portion of cellular energy (41). It is estimated that up to approximately 50% of ATP is used to support the actin cytoskeleton in a migrating cell (32). Cytoskeletal elongation forms actin-rich lamellipodia and/or filopodia at the leading edge, which are key structures for cell migration (27). We observed that durotactic lung fibroblasts reposition their mitochondria in lamellipodia/filopodia where the energy-consuming cell motor localizes. These findings suggest that human lung fibroblasts concentrate their energy production in the sites with high energy demand to support cytoskeletal activity and durotactic cell migration. We found that stiff matrix promotes ATP production that is independent of mitochondrial fission. Stiff matrix–dependent mitochondrial fission may facilitate the transport of mitochondria to lamellipodia and filopodia, where stiff matrix–induced ATP production fuels cells to generate traction forces for durotactic migration.

Mitochondria supply energy by the highly efficient OXPHOS. Most lung cells depend on aerobic glycolysis to supply carbon in the form of pyruvate to mitochondria, which support OXPHOS for production of ATP (42). Recent studies have shown that cellular bioenergetics change in pulmonary fibrosis. Glycolysis, particularly aerobic glycolysis, is elevated in fibrotic fibroblasts (43, 44). Disordered gas exchange in the fibrotic lungs decreases oxygen tension, which may force lung fibroblasts to rely on glycolysis for ATP production. While migratory cells generally favor mitochondrial respiration for increased ATP production (45), our findings suggest that both OXPHOS and glycolysis are involved in lung fibroblast durotaxis. Although the energetic yield through glycolysis is very low compared with OXPHOS, a trade-off is that ATP synthesis by glycolysis is up to 100 times quicker than through OXPHOS (46). During lung fibroblast durotaxis, glycolysis would be responsible for rapid energy requirements and OXPHOS for persistent energy needs. It has been shown that during collective migration of epithelial cells, leader cells require more energy than followers (47), probably because leader cells are responsible for a large part of the pushing and pulling forces required for cell migration. When in the leader position, cells increase glucose uptake, which elevates energy production probably by transient upregulation of glycolysis (48). Although it is currently not known whether activated/proliferative fibroblasts can migrate as a group similar to collectively migrating epithelial cells, neighboring myofibroblasts have been found to communicate through adhesion structures such as the adherens junction (49). Glycolysis might function to provide additional energy needs for leader lung fibroblasts to initiate cell group migration during durotaxis.

There are some limitations in this study. First, deeper mechanistic studies are needed to unveil the exact mechanotransduction pathway(s) by which matrix stiffness regulates mitochondrial dynamics. The mechanosensitive signals that mediate upregulation of DRP1 by stiff matrix remain unknown. Previous studies have shown that increasing ECM stiffness enhances [Ca^2+^]_i_ oscillation activity in myofibroblasts (50). Moreover, calcium regulates mitochondrial fission by calcineurin-dependent DRP1 activation (51). Thus, calcium signaling could be a good candidate that acts upstream in the mitochondrial involvement of human lung fibroblast durotaxis. Second, despite that studies utilizing stiffness gradient 2D matrices recapitulate important aspects of lung fibroblast biology relevant to native lung tissues, it is also known that additional physical properties of the ECM, such as dimensionality and topography, have important impacts on fibroblast response to matrix stiffness (52). Studying fibroblast durotaxis in 3D matrix systems will offer critical insights more relevant to physiological conditions. Last, lung mitochondria can utilize other energy sources, such as fatty acids and glutamate, for ATP production (42). A broader investigation into energy metabolism is required to fully understand mitochondrial function in lung fibroblast durotaxis.

Matrix stiffness has a profound effect on fibroblast activation in lung fibrosis. In this study, we demonstrated that stiffened matrix altered mitochondrial dynamics, ATP production, and subcellular localization in migrating human lung fibroblasts. Our study identified a mechanism through which mitochondria may contribute to the development and/or progression of fibrotic lung diseases. It highlights an abnormal mechanical environment in mitochondrial dysfunction and the role of mitochondrial dysfunction in human lung fibroblast durotaxis. Currently, mitochondrial structure and function in lung mechanobiology are underappreciated. Understanding how lung fibroblasts migrate in response to matrix mechanical cues under the disease state has the potential to lead to the identification of novel therapeutic avenues. Inhibition of durotactic migration of human lung fibroblasts may play an important role in preventing the progression of IPF.

## Methods

### Lung fibroblast isolation, culture, and transfection.

Human lung fibroblasts were established from 5 donors whose tissues were rejected for lung transplantation. Lung fibroblast isolation, culture, and transfection are described in the Supplemental Methods.

### Preparation of PA hydrogels with varying stiffness.

PA gels with Young’s modulus of 1 kPa or 20 kPa were fabricated as described in our previous studies (6, 53). Gel surfaces were coated with 0.1 mg/mL rat tail collagen I (Thermo Fisher Scientific).

PA gels with gradient stiffness mimicking the gradient strength of physiological lungs were fabricated by modifications of a previously published protocol (26). Briefly, 24 × 20 × 1 mm glass molds were generated by affixing glass slides and coverslips with superglue. A 300 μL volume of gel solution consisting of a 4% acrylamide monomer, 0.4% *N,N*′-methylenebisacrylamide crosslinker, and 0.4% collagen I was poured into dichlorodimethylsilane-treated glass molds. The gel solution was covered with a glass coverslip, which was pretreated with 0.1 M NaOH, 3-Aminopropyltriethoxysilane, and 0.5% glutaraldehyde, in a way that the polymerization chamber assumed the shape of a right-angled ramp with a 3° angle in the vertical plane. After polymerization and removal of the PA gel along with the coverslip from the mold, a second 300 μL aliquot of 6% acrylamide was poured into the mold. The gel solution was covered with the first PA gel so that the coverslip edge overhung. After polymerization of the second gel, the compound gel was removed from the mold, yielding a 24 × 20 × 1 mm PA hydrogel composed of 2 sequentially polymerized, inversely oriented, ramp-shaped gel structures. Gels were rinsed and stored in 1× PBS without Mg^2+^ and Ca^2+^ for further use.

### Mechanical testing of the PA hydrogels with gradient stiffness.

Stiffness gradient PA gels were mechanically characterized using an MFP-3D-BIO Atomic Force Microscope (Asylum Research), mounted on a Nikon Eclipse Ti2 microscope, in contact mode. Detailed AFM methods are provided in the Supplemental Methods.

### Human lung fibroblast migration on stiffness gradient PA hydrogels.

Serum-starved human lung fibroblasts were seeded in a circled area at the 2 kPa region on stiffness gradient PA gels for 2 hours to allow cell attachment. The initial position of attached cell population was recorded under a Nikon Eclipse TS2 microscope equipped with a DS-Fi3 camera. Cells were cultured in the presence of 0.2% FBS and allowed to migrate on the stiffness gradient gels. After 7 days, the position of the cell population was recorded again. The geometric centers (centroids), defined as the arithmetic mean position of all the points in a 2D shape, of lung fibroblast population at 2 hours and 7 days were identified with Matlab R2019a (MathWorks, Inc.) and were used to calculate the overall distance of lung fibroblast migration. Durotactic migration was calculated as the distance in the direction of increasing stiffness.

### Confocal laser scanning microscopy.

Fluorescent signals were detected using a confocal laser scanning microscope Zeiss LSM510 confocal microscope equipped with a digital color camera. All fluorescent images were generated using sequential laser scanning with only the corresponding single-wavelength laser line, activated using acousto-optical tunable filters to avoid cross-detection of either one of the fluorescence channels.

### Animals and experimental protocol.

Animal usage and bleomycin protocols were approved by the Institutional Animal Care and Use Committee of the University of Alabama at Birmingham. Six- to 8-week-old pathogen-free female mice (C57BL/6, The Jackson Laboratory) were used in this study. Bleomycin sulfate (Almirall) was dissolved in 50 μL sterile saline solution and administered in mice by oropharyngeal aspiration at a single dose of 2 U/kg body weight. Control mice received an equal volume of saline. Mice were sacrificed at 21 days. Lung tissues were excised and inflated with OCT for immunofluorescence or formalin-fixed and embedded in paraffin for histological analysis.

To prepare lung homogenates, fresh lung tissues were transferred to prechilled tubes containing T-PER and Complete Mini Protease Inhibitor Cocktail (Thermo Fisher Scientific), at a proportion of 1 tablet/10 mL of T-PER, and were homogenized at 4°C. Homogenates were centrifuged at 9,000*g* for 10 minutes at 4°C. Supernatants were transferred to clean microcentrifuge tubes. Total protein concentrations in the lung tissue homogenates were determined using a BCA Protein Assay Kit (Thermo Fisher Scientific).

### Statistics.

Statistical differences among treatment conditions were determined by 1-way ANOVA (Newman-Keuls method for multiple comparisons) or 2-tailed Student’s *t* test using GraphPad Prism 8 software. Pearson’s correlation coefficient analysis was used to measure the association between variables of interest. Values of *P* < 0.05 were considered significant.

### Study approval.

The animal studies were conducted in accordance with the NIH *Guide for Care and Use of Laboratory Animals* (National Academies Press, 2011) and approved by the Institutional Animal Care and Use Committee at the University of Alabama at Birmingham. The studies involving human participants were approved by Institutional Review Board at the University of Alabama at Birmingham. Participants have provided written informed consent.

Additional detail on the methods is provided in the online Supplemental Methods.

## Author contributions

TG and Y Zhou designed the study; TG, CJ, SZY, Y Zhu, and CH performed experiments; TG, Y Zhu, and Y Zhou analyzed data; ABC, VBA, and HP participated in discussion; and TG and Y Zhou wrote the manuscript.

## Supplementary Material

Supplemental data

Supplemental video 1

Supplemental video 2

Supplemental video 3

Supplemental video 4

Supplemental video 5

Supplemental video 6

Supplemental video 7

## Version 1 11/24/2022

In-Press Preview

## Version 2 01/10/2023

Electronic publication
