# Supplementary material for: Mitochondrial fission and bioenergetics mediate human lung fibroblast durotaxis
Source: JCI Insight. 2023 Jan 10;8(1):e157348. doi: 10.1172/jci.insight.157348 (PMC9870082; doi:10.1172/jci.insight.157348)
Supplement: Supplemental data [file jciinsight-8-157348-s028.pdf]

# Mitochondrial Fission and Bioenergetics Mediate Human Lung Fibroblast Durotaxis

Ting Guo[1,2], Chun-sun Jiang[1], Shan-Zhong Yang[1], Yi Zhu[1], Chao He[1], A. Brent Carter[1], Veena B. Antony[1], Hong Peng[2], and Yong Zhou[1,*]

Online Supplemental Materials

## SUPPLEMENTAL METHODS

### Antibodies and reagents

Anti-Drp1, anti-MFF, and anti-FIS1 antibodies were purchased from Proteintech (Rosemont, IL). Anti-GAPDH antibodies were from Santa Cruz Biotechnology (Santa Cruz, CA). Anti-collagen I alpha I antibodies and anti-Npnt antibodies were from Fisher Scientific (Hampton, NH). Anti-αSMA antibody was from American Research Products (Waltham, MA). 2-Deoxy-D-glucose (2-DG) was from Thermo Fisher (ThermoFisher Scientific, Waltham, MA, USA). Oligomycin A was from Selleckchem (Selleck Chemicals, Houston, TX, USA). P259 and TAT-carrier control peptides were provided Dr. Daria Mochly-Rosen (1). P259 or TAT-carrier control treatment of fibroblasts was carried out in the growth medium every 24 hours. DRP1 siRNAs and scrambled siRNAs were from Origene (Rockville, MD, USA). Plasmids encoding mCherry-tagged DRP1 and mCherry alone were purchased from Addgene (Watertown, MA, USA).

### Isolation, culture, transfection, and treatment of primary human lung fibroblasts

Primary human lung fibroblasts were established from 5 donors whose tissues were rejected for lung transplantation. Lungs were minced in sterile phosphate-buffered saline and tissue pieces were placed in 100-mm tissue culture dishes containing Dulbecco's modified Eagle's medium (DMEM), 10% fetal bovine serum (FBS), 1% penicillin/streptomycin/ glutamine, nonessential amino acids, and sodium pyruvate (supplemented DMEM). Medium was replenished every 3 d. After 14 d, cells growing out of the explants were trypsinized and plated in supplemented DMEM. Lung fibroblasts were used between passages 6 and 10. All three fibroblast populations maintain normal cell appearance and grow at a stable speed for a period of at least 15 cell passages. Lung fibroblasts were cultured on 1 kPa or 20 kPa PA gels in the presence of 2% FBS.

For cell migration assay on stiffness-gradient gels, lung fibroblasts were serum-starved for 24 hours and then were seeded on stiffness-gradients gels in the presence or absence of 2 μM p259 or TAT-carrier control peptides, 1 mM 2-DG, and 5 μM Oligomycin A. To reduce fibroblast proliferation and aid cell migration, 0.5% FBS were added to lung fibroblasts cultured on stiffness-gradient gels. Plasmids or siRNAs were transfected into 1 x $10^6$ lung fibroblasts by a Nucleofector 2b device (Lonza, Basel, Switzerland) according to the manufacturer's protocol. Briefly, cell pellets were resuspended in 100 μl room-temperature Nucleofector Solution R and 2 μg DNA or 100 nM siRNA. Cell suspension was transferred into a cuvette, and electroporation was carried out using Nucleofector Program V-001. 500 μl of the pre-equilibrated culture medium was immediately added to the cuvette. Transfected cells were gently transferred to the prepared hydrogels and cultured until harvesting.

**Quantitative analysis of mitochondria**

Quantifications with the Mitochondrial Analyzer were performed based on standard confocal fluorescence microscopy and the open-source image analysis platform ImageJ/Fiji as described previously (2). Briefly, confocal micrographs were processed and thresholded, and the resulting binary images were used as inputs for measuring the mitochondrial area. For network connectivity analysis, the binarized mitochondria were converted into topological skeletons. The skeleton map was used to calculate the number of branches, branch lengths, and branch junctions in the skeletonized network.

**Quantitative real-time PCR**

Total RNA was extracted with the RNeasy Mini Kit (QIAGEN, Valencia, CA, USA). 1 μg total RNA was reversely transcribed into cDNA with a cDNA Synthesis Kit (ThermoFisher Scientific, Waltham, MA). Quantitative PCR reactions were carried out in a Bio-Rad iCycler (Bio-Rad Laboratories, Hercules, CA). Each sample was run in triplicate. Relative quantification was calculated using the comparative $C_T$ method. Delta CT values of target gene were normalized to GAPDH and subjected to statistical analysis. The ratio of distal transcripts/common transcripts was calculated as $\Delta CT = (CT_{distal} - CT_{GAPDH}) - (CT_{common} - CT_{GAPDH}) = CT_{distal} - CT_{common}$.

**Immunoblot and densitometry analysis**

Cell lysates containing 10 - 20 μg total proteins were loaded onto multiple SDS-polyacrylamide gels under reducing conditions. Each gel loaded with the same samples with the same amount of cell lysates was used for detection of a specific target protein. After electrophoresis, proteins were electrophoretically transferred from the gels to nitrocellulose at 100 V for 1.5 hr at 4°C. Membranes were blocked in casein solution (1% casein, 25 mM $Na_2HPO_4$, pH 7.1) for 1 hr at room temperature. Primary antibodies were diluted in TBS-T and casein solution (1:1) at a working concentration recommended by manufactures. Membranes were incubated with primary antibodies at room temperature for 1 hr. After extensive washing, membranes were incubated with peroxidase-conjugated secondary antibodies (0.1 μg/ml) diluted in TBS-T for 1 hr at room temperature. Immunodetection was carried out by chemiluminescence. Blot images were scanned. Bands were quantified by ImageJ (NIH, Bethesda).

**Transwell migration assays**

Lung fibroblasts on soft (1 kPa) and stiff (20 kPa) PA gels were detached by trypsinization. An equal number of living cells (1 x $10^5$ cells per chamber) were suspended in 500 µl of serum-free medium and then plated in the upper chamber of transwell inserts that feature a tissue culture-treated polyester (PET) membrane for optimal cell attachment (VWR, Radnor, PA, USA). Culture medium with 10% FBS used as chemoattractant was added to the lower chambers. Cells were cultured in a humidified incubator at 37°C with 5% $CO_2$. Cell migration was measured at 7 hours to minimize the potential effect of differential matrix stiffness on fibroblasts proliferation. Transwell inserts were washed twice with phosphate-buffered saline. Cells on the inside of the transwell inserts were gently removed using moistened cotton swabs, and cells on the lower surface of the membrane were then stained with 0.5% crystal violet for 30 min. The transwell inserts were washed twice with PBS to remove unbound crystal violet and then air-dried. The migrated cells were observed and imaged under a Nikon Eclipse TS2 microscope equipped with DS-Fi3 camera. Inserts were eluted in 400 µl 33% acetic acid for 10 min. The eluents were transferred to a 96-well microplate and absorbance was measured at 590 nm using a plate reader. Cell number was calculated based on a standard curve plotted from a series of numbers of human lung fibroblasts that migrated on transwells.

**Measurements of ATP levels**

ATP levels were measured using a luminescent ATP detection assay kit according to the manufacturer's protocol (Cayman, Ann Arbor, MI, USA). Luminescence was measured using a microplate reader (Molecular Devices, Sunnyvale, CA, USA). The ATP concentration was

determined by interpolation within the ATP standard reference. The relative ATP levels were corrected by total DNA.

**Mechanical testing by AFM microindentation**

The AFM system was calibrated according to the manufacturer's instruction before each indentation measurement, and the cantilever spring constant was confirmed by the thermal fluctuation method. Force-indentation profiles were acquired at an indentation rate of 20 μm/s separated by 4 μm spatially in a 16 × 16 sample grid covering a 20 × 20–μm area. The elasticity (Young's modulus) at each point on the grid was calculated from fitting force-indentation data using a Hertz-based model: $F=2E\delta^2\tan(\alpha)/\pi(1-\nu^2)$, where indentation force ($F$) was calculated by using Hooke's law ($F = \kappa\Delta x$), where κ and $\Delta x$ denote the AFM probe's spring constant and the probe's measured deflection, respectively. The indentation depth ($\delta$) is calculated from the difference in the $z$-movement of the AFM piezo and the deflection of the probe. E is the elastic modulus of lung tissues being studied, and ν denotes the Poisson ratio (0.4). $\alpha$ represents the shape of the probes that were considered to be conical with an approximated half-angle of 35 degrees in this report. Stiffness-gradient PA gels were indented at 5 mm intervals in the direction of increasing stiffness. A contour map was plotted to visualize spatial patterns of elasticity collected in Force-Map mode using the Matlab R2019a (MathWorks, Inc., Natick, MA).

**Flow cytometry**

Single cell suspensions were pelleted and washed with PBS three times. Flow cytometry was performed on a LSRII Flow Cytometer (BD Biosciences, San Jose, CA). Data were processed using FACSDiva software (BD Biosciences).

**Confocal fluorescence microscopy and time-lapse microscopy**

Cells cultured on PA gels were loaded with 50nM MitoTracker Red CMXRos (Invitrogen, Eugene, OR, USA) for 45 min to stain mitochondria. Then, cells were fixed with 3.7% formaldehyde, permeabilized with 0.5% Triton X-100, and incubated with phalloidin CF488A (Biotium, Fremont, CA, USA) to stain actin filaments. Nuclei were stained with DAPI (Thermo Fisher Scientific, Waltham, MA). Fluorescent signals were detected using a confocal laser-scanning microscope Zeiss LSM510 confocal microscope (Oberkochen, Germany) equipped with a digital color camera (Oberkochen, Germany). All fluorescent images were generated using sequential laser scanning with only the corresponding single wavelength laser line, activated using acousto-optical tunable filters to avoid cross-detection of either one of the fluorescence channels. The area of mitochondria was measured using ImageJ (NIH, Bethesda). Time-lapse imaging of single lung fibroblast migration on stiffness-gradient gels was conducted using a Lionheart FX automated imaging system from BioTek (Winooski, VT), with images collected at 30-min intervals for 24 hours at 37°C and 5% $CO_2$. Images were acquired using the Gen5 software, and the resulting pictures were further handled with ImageJ.

**Lung histology and immunofluorescent staining**

Masson trichrome staining was performed to evaluate collagen deposition in mouse lung tissues according to the manufacturer's recommendation (Polysciences Inc, Warrington, PA, USA). Digital images of the stained sections were captured using a Nikon Eclipse TS2 microscope equipped with DS-Fi3 camera.

30 μm cryostat sections were rehydrated in PBS for 10 minutes. Tissue sections were blocked with 5% normal goat serum and co-stained with anti-αSMA (1:200 dilutions), anti-NPNT (1:200 dilutions), anti-DRP1 (1:100 dilutions) or anti-MFF (1:100 dilutions) antibodies diluted in PBS containing 1% goat serum, 0.3% Triton X-100, and 0.01% sodium azide according to manufacturer's instructions. Fluorochrome-conjugated secondary antibodies (SouthernBiotech, Birmingham, AL) were used according to the manufacturer's recommendation. Nuclei were stained with DAPI (Thermo Fisher Scientific, Waltham, MA). Fluorescent signals were detected using a confocal laser-scanning microscope Zeiss LSM710 confocal microscope equipped with a digital color camera (Oberkochen, Germany). Fluorescence intensity was quantified by ImageJ.

## SUPPLEMENTAL FIGURE LEGENDS

Supplemental Figure 1: **Evaluation of mitochondrial mass by flow cytometry analysis**. Human lung fibroblasts cultured on soft and stiff PA gels were stained by Mitrotracker Green. The intensity of green fluorescence, a measure of mitochondrial mass (3, 4), was determined by flow cytometry analysis. Results are mean ± SD of three independent experiments. A two-tailed Student's t test was used for comparison between groups.

Supplemental Figure 2: **Effects of DRP1 knockdown on mitochondrial connectivity in human lung fibroblasts cultured under soft and stiff matrix conditions**. **A** and **B**, Primary human lung fibroblasts were cultured on soft (A) and stiff (B) PA gels in the presence or absence of DRP1 siRNAs or the control siRNAs. Mitochondria and DRP1 were stained by MitoTracker (red) and anti-DRP1 antibody (green), respectively. The binarized mitochondria were converted into topological skeletons. Scale bar = 20 μm; **C**, The number of branches, length of branches, and mean branch length in the skeletonized mitochondrial network were measured by Mitochondria Analyzer. Bar graphs represent mean ± SD per cell from total 30 individual cells from 3 human subjects (n = 10 cells per subject) under each condition. Statistical analysis was performed by one-way ANOVA.

Supplemental Figure 3: **Effects of DRP1 overexpression on mitochondrial connectivity in human lung fibroblasts cultured under soft and stiff matrix conditions**. **A** and **B**, Primary human lung fibroblasts were transfected by DRP1-mCherry-expressing or mCherry-expressing vectors by a Nucleofector 2b device. Cells were then cultured on soft (A) and stiff (B) PA gels. Mitochondria were stained by MitoTracker (green). The binarized mitochondria were converted into topological skeletons. Scale bar = 20 μm; C, The number of branches,

length of branches, and mean branch length in the skeletonized mitochondrial network were measured by Mitochondria Analyzer. Bar graphs represent mean ± SD per cell from total 30 individual cells isolated from 3 independent human subjects ( n = 10 cells per subject) under each condition. Statistical analysis was performed by one-way ANOVA.

Supplemental Figure 4: **Evaluation of cell attachment and viability of pre-conditioned human lung fibroblasts in transwells**. Human lung fibroblasts were cultured on soft and stiff PA gels for 72 hours. Pre-conditioned cells were detached from PA gels by trypsin. An equal number of pre-conditioned living lung cells were seeded in transwells and incubated for 2 hours. Cell attachment and viability were evaluated by crystal violet staining followed by colorimetric assays. Results are mean ± SD of three independent experiments. A two-tailed Student's t test was used for comparison between groups.

Supplemental Figure 5: **Fabrication and characterization of stiffness-gradient hydrogels simulating the rigidity of physiological lungs. A**, Schematic and pictorial illustrations of the fabrication of stiffness-gradient PA hydrogels and characterization of the mechanical properties of PA gels by AFM microindentation. **B**, Heat maps show the elastic moduli at the selected regions on the 6%/4% gradient gels. **C**, The dot plot graph shows the Young's moduli at the selected regions. The horizontal lines indicate median Young's moduli at each region. **D**, Linear regression analysis reveals that the gradient strength of PA gels is 829.4 Pa/μm ($R^2 = 0.8447$).

Supplemental Figure 6: **Tracking single human lung fibroblast migration on stiffness-gradient hydrogels by time-lapse microscopy**. **A**, Primary human lung fibroblasts were cultured on stiffness-gradient PA gels. Single cell movement was recorded for 24 hours by a Lionheart FX automated microscope. Scale bar = 1000 μm; **B**, Cell tracking and trajectory analysis was performed using the MTrackJ plugin in ImageJ. The distance of durotactic migration (231 μm) was determined as described previously.

Supplemental Figure 7: **Effects of p259 peptides on ATP production**. Primary human lung fibroblasts were cultured on soft and stiff PA gels in the presence of p259 peptides or the control TAT peptides. The levels of ATP produced by cells were determined by luminescent ATP detection assays. Results were corrected by total DNA. Bar graphs represent mean ± SD of three independent experiments. Statistical analysis was performed by one-way ANOVA.

Supplemental Figure 8: **Effects of p259 peptides, oligomycin, and 2DG on single human lung fibroblast durotaxis**. Primary human lung fibroblasts were cultured on stiffness-gradient PA gels in the presence of p259 peptides or the control TAT peptides (A), Oligomycin or DMSO (vehicle for Oligomycin ) (B), and 2DG or PBS (vehicle for 2DG) (C). Single cell movement was recorded for 24 hours by a Lionheart FX automated microscope. Cell tracking and trajectory analyses were performed using the MTrackJ plugin in ImageJ. The distance of durotactic cell migration under each condition was determined as described previously. Bar graphs represent mean ± SD per cell from 30 individual cells isolated from 3 independent human subjects (n = 10 cells per subject) under each condition. Scale bar = 1000 μm. A two-tailed Student's t test was used for comparison between groups.

Supplemental Figure 9: **Evaluation of DRP1 and MFF expression in (myo)fibroblasts in normal and fibrotic lungs of human and mice**. Frozen lung tissue sections from normal and fibrotic human and mouse lungs were subjected to co-immunofluorescence staining of DRP1/Drp1, MFF/Mff, and normal fibroblast marker (NPNT/Npnt) and myofibroblast marker (αSMA/αSma). Co-localization of DRP1/MFF expression with (myo)fibroblasts was evaluated by Pearson's correlation coefficient analyses. Scale bar = 20 μm.

## SUPPLEMENTAL REFERENCES


1. Kornfeld OS, Qvit N, Haileselassie B, Shamloo M, Bernardi P, and Mochly-Rosen D. Interaction of mitochondrial fission factor with dynamin related protein 1 governs physiological mitochondrial function in vivo. *Sci Rep.* 2018;8(1):14034.
2. Chaudhry A, Shi R, and Luciani DS. A pipeline for multidimensional confocal analysis of mitochondrial morphology, function, and dynamics in pancreatic β-cells. *Am J Physiol Endocrinol Metab.* 2020;318(2):E87-E101.
3. Doherty E, and Perl A. Measurement of Mitochondrial Mass by Flow Cytometry during Oxidative Stress. *React Oxyg Species (Apex).* 2017;4(10):275-83.
4. Monteiro LB, Davanzo GG, de Aguiar CF, and Moraes-Vieira PMM. Using flow cytometry for mitochondrial assays. *MethodsX.* 2020;7:100938.

Supplemental Figure 1

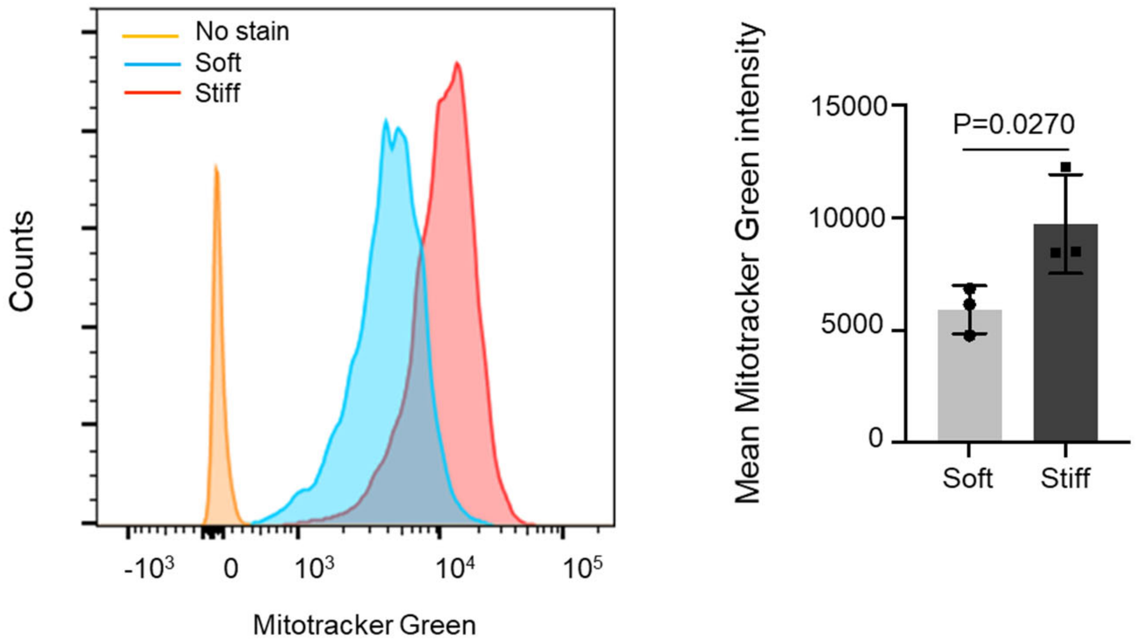


Supplemental Figure 1: **Evaluation of mitochondrial mass by flow cytometry analysis**. Human lung fibroblasts cultured on soft and stiff PA gels were stained by MitroTracker Green. The intensity of green fluorescence, a measure of mitochondrial mass (3, 4), was determined by flow cytometry analysis. Results are mean ± SD of three independent experiments. A two-tailed Student's t test was used for comparison between groups.

Supplemental Figure 2

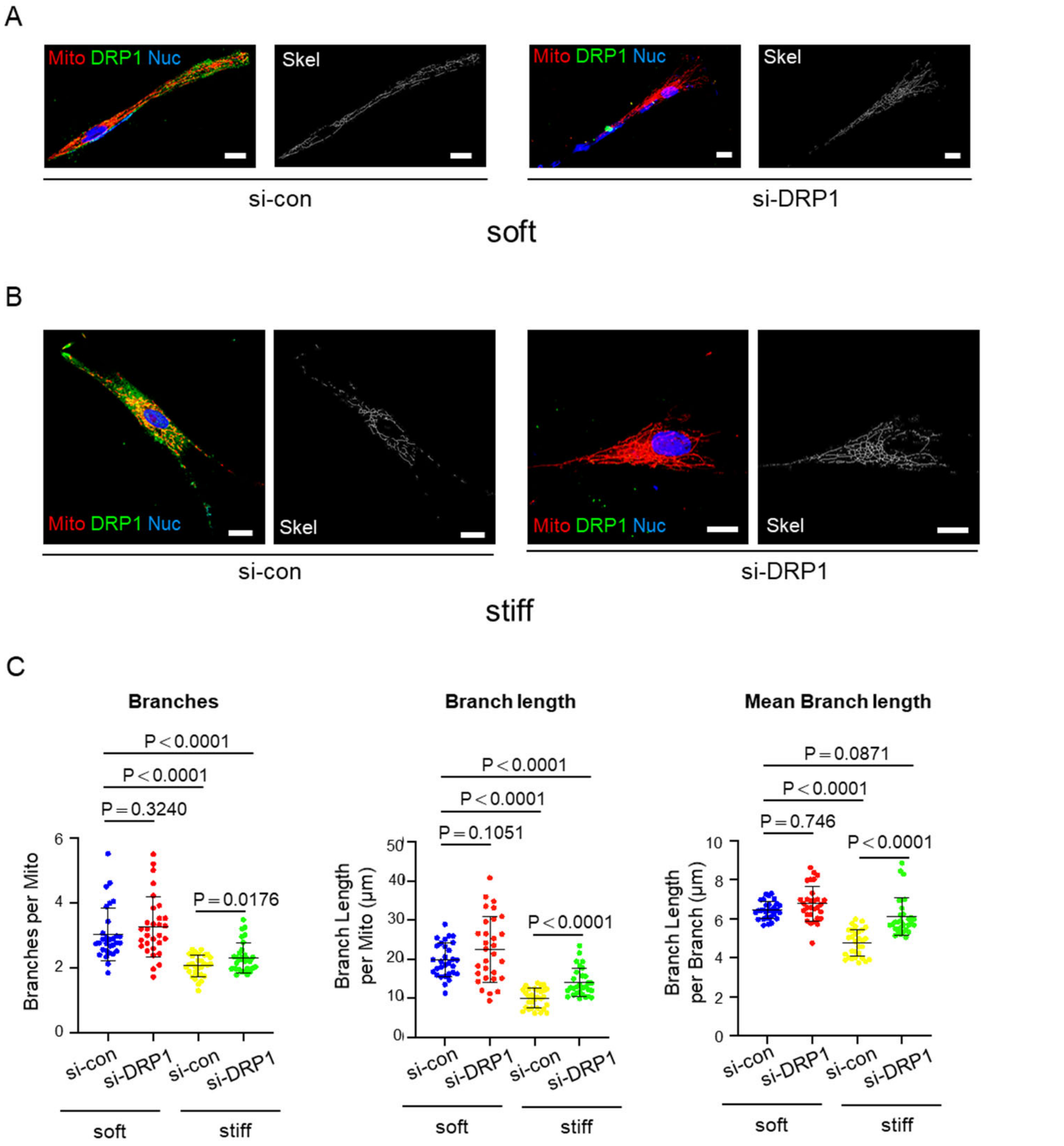


Supplemental Figure 2: **Effects of DRP1 knockdown on mitochondrial connectivity in human lung fibroblasts cultured under soft and stiff matrix conditions**. **A** and **B**, Primary human lung fibroblasts were cultured on soft (A) and stiff (B) PA gels in the presence or absence of DRP1 siRNAs or the control siRNAs. Mitochondria and DRP1 were stained by MitoTracker (red) and anti-DRP1 antibody (green), respectively. The binarized mitochondria were converted into topological skeletons. Scale bar = 20 μm; **C**, The number of branches, length of branches, and mean branch length in the skeletonized mitochondrial network were measured by Mitochondria Analyzer. Bar graphs represent mean ± SD per cell from total 30 individual cells from 3 human subjects (n = 10 cells per subject) under each condition. Statistical analysis was performed by one-way ANOVA.

Supplemental Figure 3

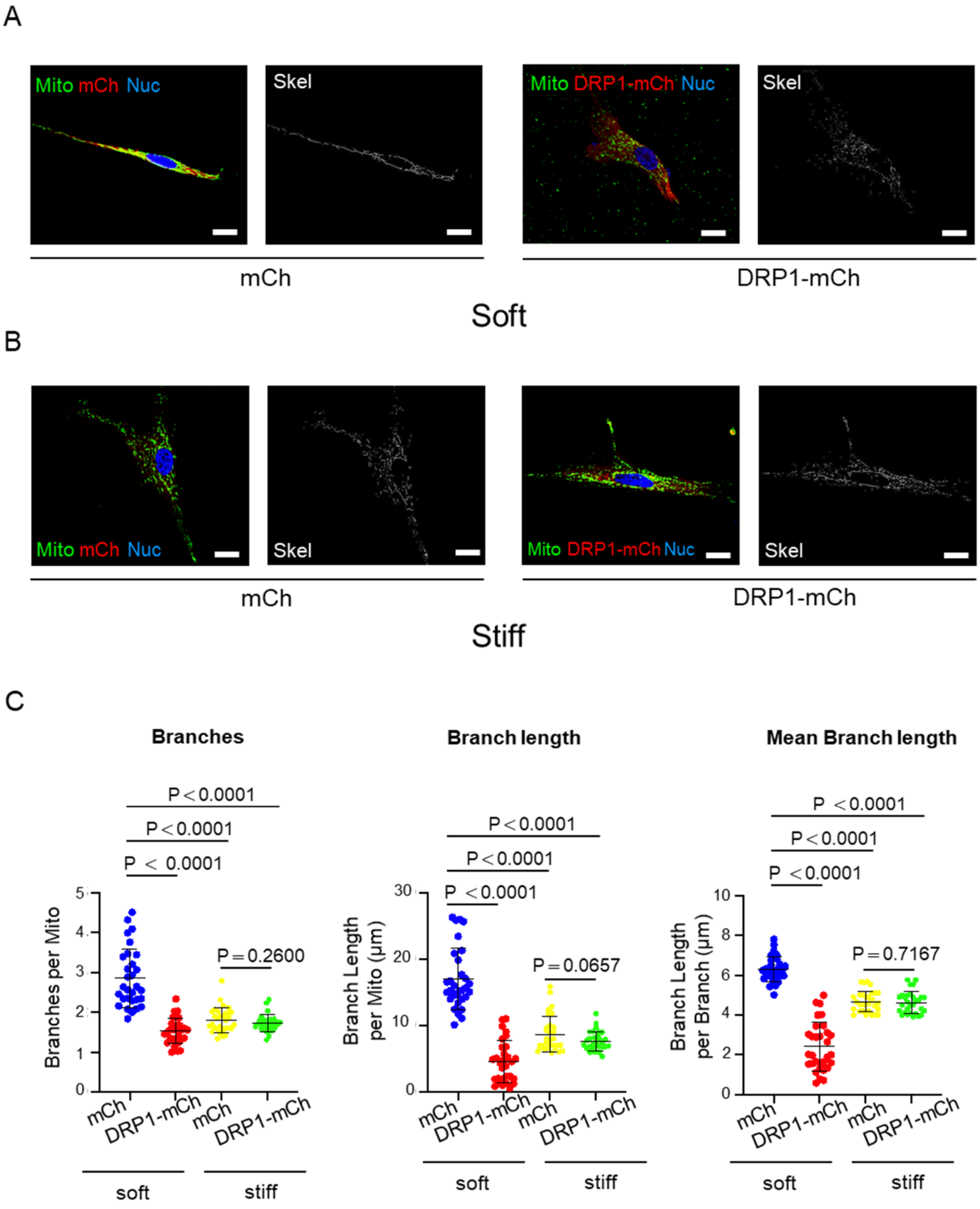


Supplemental Figure 3: **Effects of DRP1 overexpression on mitochondrial connectivity in human lung fibroblasts cultured under soft and stiff matrix conditions**. **A** and **B**, Primary human lung fibroblasts were transfected by DRP1-mCherry-expressing or mCherry-expressing vectors by a Nucleofector 2b device. Cells were then cultured on soft (A) and stiff (B) PA gels. Mitochondria were stained by MitoTracker (green). The binarized mitochondria were converted into topological skeletons. Scale bar = 20 µm; **C**, The number of branches, length of branches, and mean branch length in the skeletonized mitochondrial network were measured by Mitochondria Analyzer. Bar graphs represent mean ± SD per cell from total 30 individual cells isolated from 3 independent human subjects ( n = 10 cells per subject) under each condition. Statistical analysis was performed by one-way ANOVA.

Supplemental Figure 4

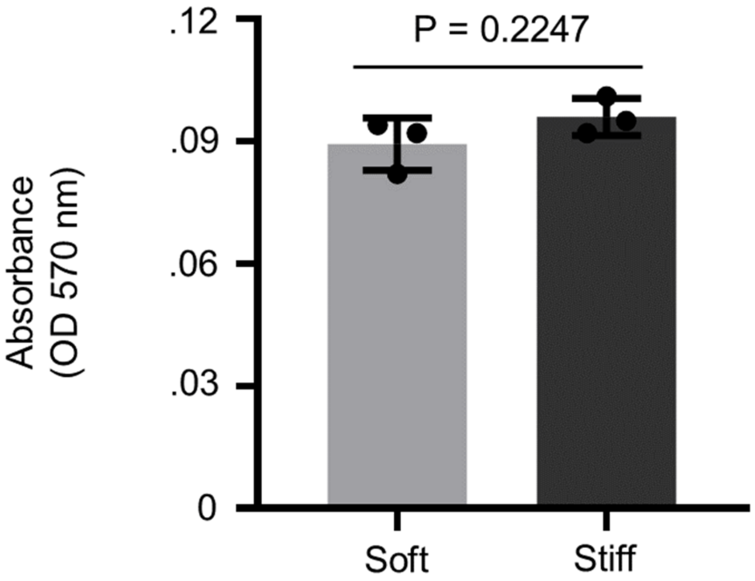


Supplemental Figure 4: **Evaluation of cell attachment and viability of pre-conditioned human lung fibroblasts in transwells**. Human lung fibroblasts were cultured on soft and stiff PA gels for 72 hours. Pre-conditioned cells were detached from PA gels by trypsin. An equal number of pre-conditioned living lung cells were seeded in transwells and incubated for 2 hours. Cell attachment and viability were evaluated by crystal violet staining followed by colorimetric assays. Results are mean ± SD of three independent experiments. A two-tailed Student's t test was used for comparison between groups.

Supplemental Figure 5

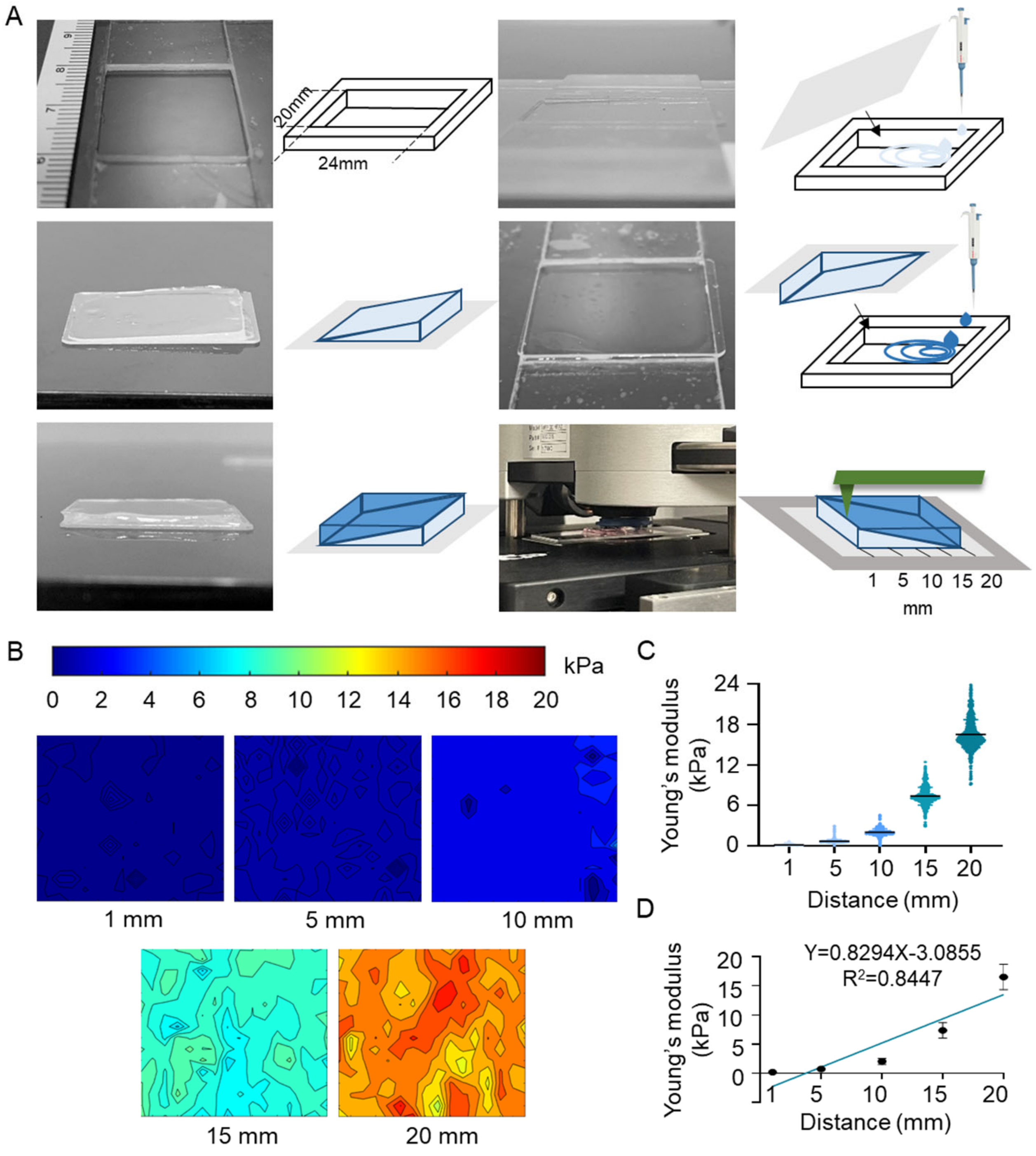


Supplemental Figure 5: **Fabrication and characterization of stiffness-gradient hydrogels simulating the rigidity of physiological lungs. A**, Schematic and pictorial illustrations of the fabrication of stiffness-gradient PA hydrogels and characterization of the mechanical properties of PA gels by AFM microindentation. **B**, Heat maps show the elastic moduli at the selected regions on the 6%/4% gradient gels. **C**, The dot plot graph shows the Young's moduli at the selected regions. The horizontal lines indicate median Young's moduli at each region. **D**, Linear regression analysis reveals that the gradient strength of PA gels is 829.4 Pa/μm ($R^2 = 0.8447$).

Supplemental Figure 6

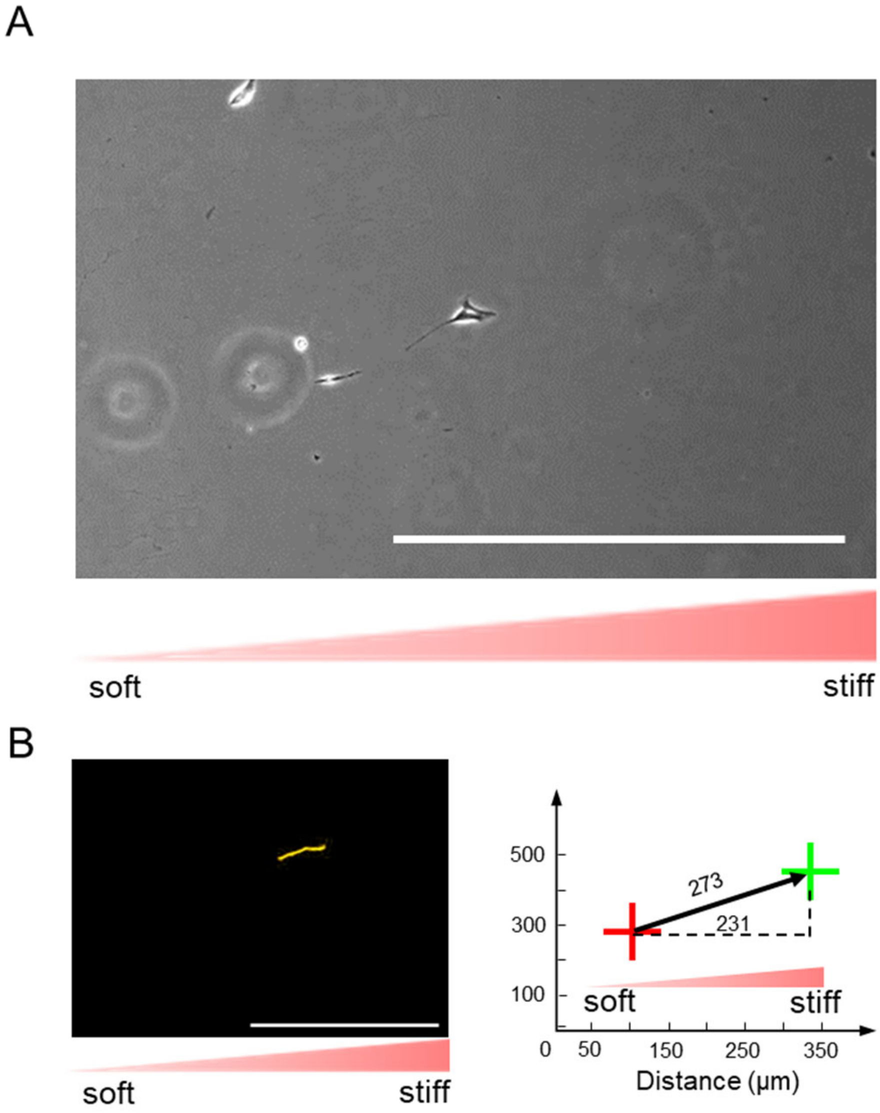


Supplemental Figure 6: **Tracking single human lung fibroblast migration on stiffness-gradient hydrogels by time-lapse microscopy**. **A**, Primary human lung fibroblasts were cultured on stiffness-gradient PA gels. Single cell movement was recorded for 24 hours by a Lionheart FX automated microscope. Scale bar = 1000 μm; **B**, Cell tracking and trajectory analysis was performed using the MTrackJ plugin in ImageJ. The distance of durotactic migration (231 μm) was determined as described previously.

Supplemental Figure 7

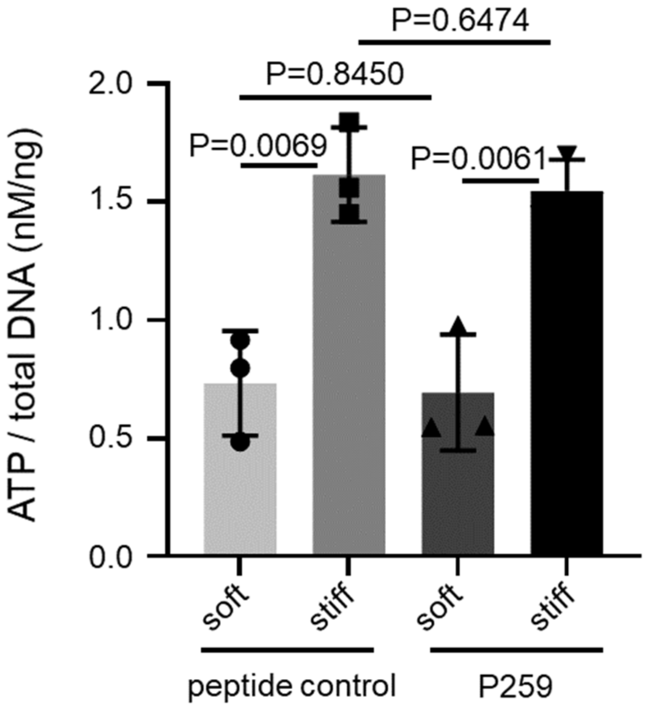


Supplemental Figure 7: **Effects of p259 peptides on ATP production**. Primary human lung fibroblasts were cultured on soft and stiff PA gels in the presence of p259 peptides or the control TAT peptides. The levels of ATP produced by cells were determined by luminescent ATP detection assays. Results were corrected by total DNA. Bar graphs represent mean ± SD of three independent experiments. Statistical analysis was performed by one-way ANOVA.

Supplemental Figure 8

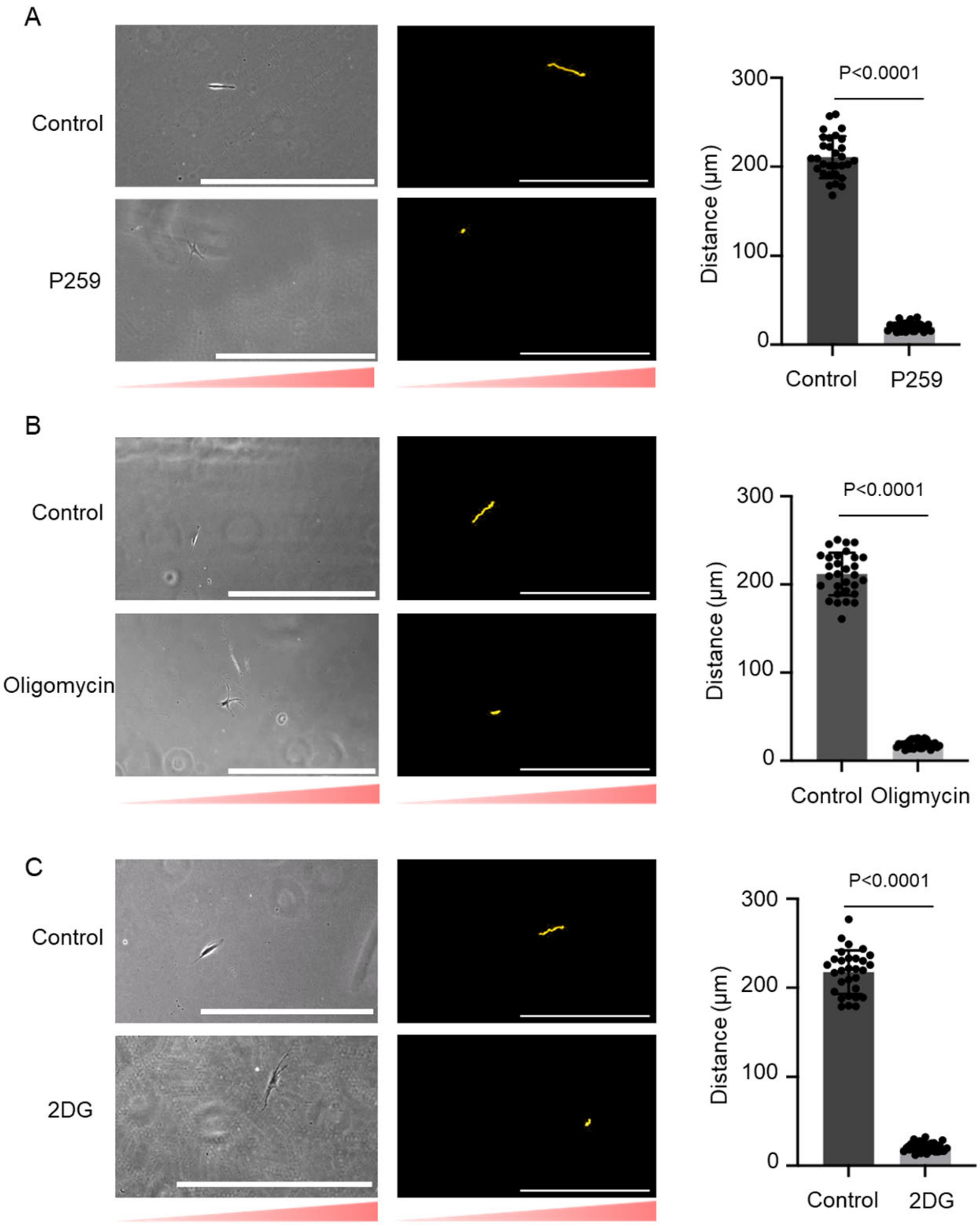


Supplemental Figure 8: **Effects of p259 peptides, oligomycin, and 2DG on single human lung fibroblast durotaxis**. Primary human lung fibroblasts were cultured on stiffness-gradient PA gels in the presence of p259 peptides or the control TAT peptides (A), Oligomycin or DMSO (vehicle for Oligomycin ) (B), and 2DG or PBS (vehicle for 2DG) (C). Single cell movement was recorded for 24 hours by a Lionheart FX automated microscope. Cell tracking and trajectory analyses were performed using the MTrackJ plugin in ImageJ. The distance of durotactic cell migration under each condition was determined as described previously. Bar graphs represent mean ± SD per cell from 30 individual cells isolated from 3 independent human subjects (n = 10 cells per subject) under each condition. Scale bar = 1000 µm; A two-tailed Student's t test was used for comparison between groups.

Supplemental Figure 9

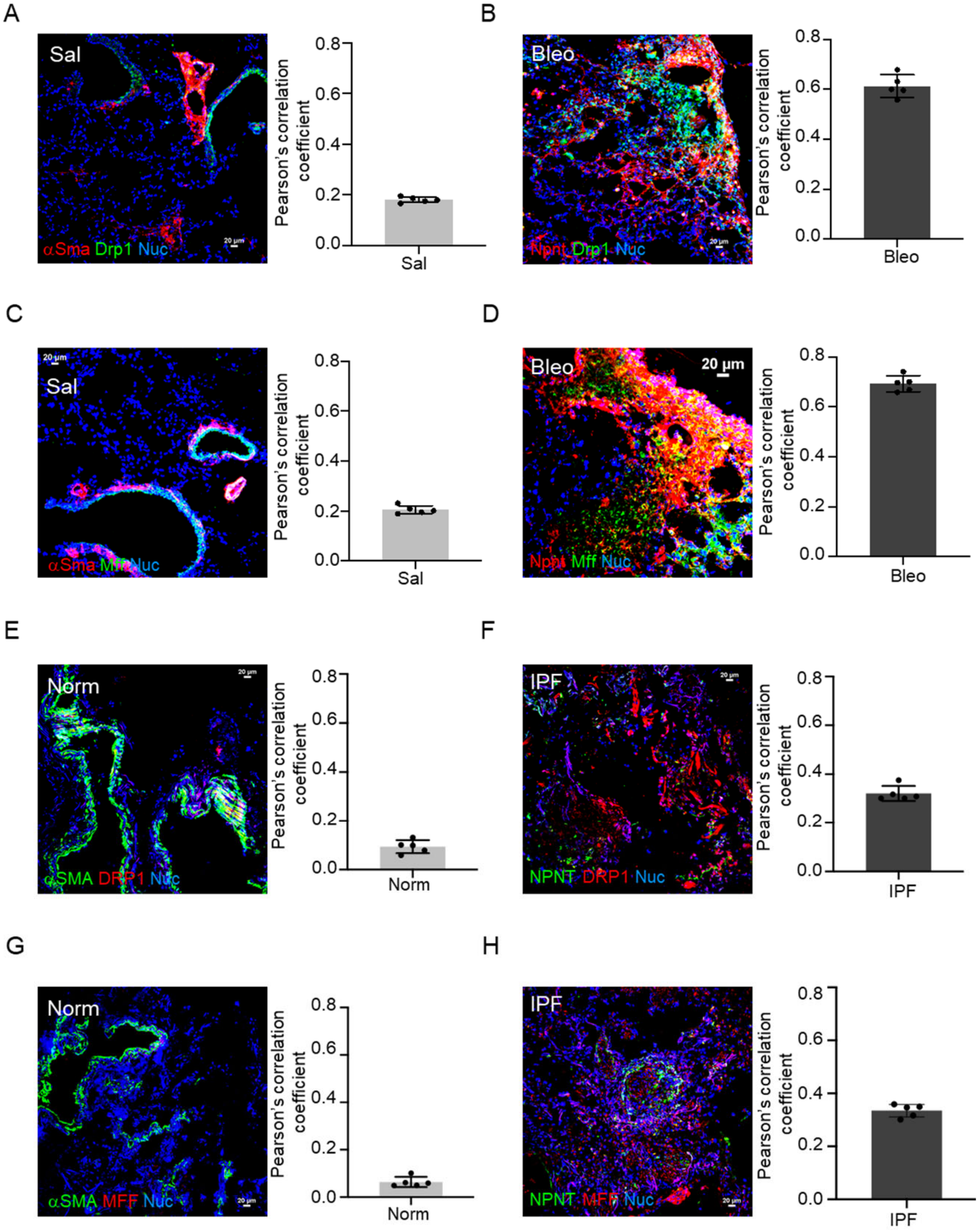


Supplemental Figure 9: **Evaluation of DRP1 and MFF expression in (myo)fibroblasts in normal and fibrotic lungs of human and mice**. Frozen lung tissue sections from normal and fibrotic human and mouse lungs were subjected to co-immunofluorescence staining of DRP1/Drp1, MFF/Mff, and normal fibroblast marker (NPNT/Npnt) and myofibroblast marker (αSMA/αSma). Co-localization of DRP1/MFF expression with (myo)fibroblasts was evaluated by Pearson's correlation coefficient analyses. Scale bar = 20 µm.
